# Cardiac Endothelial Enhancer RNA Tek‐e1 Restores Microvascular Homeostasis After Myocardial Infarction

**DOI:** 10.1002/advs.78650

**Published:** 2026-11-09

**Authors:** Yang Lyu, Kun Yang, Yunzhi Ling, Jiahui Cheng, Jiaran Shi, Shiyu Hu, Changyi Zhou, Wei Wei, Hanchuan Chen, Xiang Wei, Zengru Wang, Zhi Xiong, Chenxi Yang, Su Zou, Rui Shen, Yuanyue Song, Gaofeng Zhang, Rifeng Gao, Kaiyang Lin, Yingjia Xu

**Affiliations:** ^1^ Department of Cardiology Shanghai Fifth People's Hospital Fudan University Shanghai China; ^2^ Department of Geriatric Medicine Huashan Hospital Shanghai Medical College Fudan University Shanghai China; ^3^ Department of Cardiology The Second Affiliated Hospital Zhejiang University School of Medicine Hangzhou China; ^4^ Department of Cardiology Shengli Clinical Medical College of Fujian Medical University Fujian Provincial Hospital Fuzhou University Affiliated Provincial Hospital Fuzhou China; ^5^ Department of Radiology Renji Hospital Shanghai Jiaotong University School of Medicine Shanghai China; ^6^ Department of Cardiology The Affiliated Lihuili Hospital of Ningbo University Ningbo China; ^7^ Department of Cardiology Shanghai Institute of Cardiovascular Diseases Zhongshan Hospital Fudan University Shanghai China; ^8^ State Key Laboratory of Vascular Homeostasis and Remodeling Department of Cardiology and Institute of Vascular Medicine Peking University Third Hospital Beijing China; ^9^ Department of Hypertension Shanghai Institute of Hypertension Ruijin Hospital Shanghai Jiaotong University School of Medicine Shanghai China; ^10^ Institute of Cardiovascular Physiology and Pathophysiology Biomedical Center Ludwig‐Maximilians‐Universität München Planegg‑Martinsried Germany; ^11^ Institute of Diagnostic and Interventional Radiology Shanghai Sixth People's Hospital Shanghai Jiao Tong University School of Medicine Shanghai China; ^12^ Department of Neurosurgical Intensive Care Unit Huashan Hospital Fudan University Shanghai China; ^13^ Department of Cardiology Xinhua Hospital Affiliated to Shanghai Jiao Tong University School of Medicine Shanghai China; ^14^ Department of Cardiac Surgery The Second Affiliated Hospital Zhejiang University School of Medicine Hangzhou China

**Keywords:** endothelial epigenetic remodelling, enhancer rna, microvascular homeostasis, myocardial infarction, r‐loop

## Abstract

Microvascular repair after myocardial infarction involves both angiogenesis and re‐establishment of vascular homeostasis. Here, we identify a distinct transcriptionally active state in hypoxic border‐zone endothelial cells driven by KDM5A downregulation and biased towards endothelial stabilization and microvascular homeostatic repair. KDM5A loss increases H3K4me3 enrichment at the *Tek* promoter and an intronic enhancer, enabling ISL1 to coordinately activate transcription of *Tek* and the enhancer RNA Tek‐e1. Tek‐e1 acts as an essential cis‐regulatory RNA that further amplifies *Tek*/TIE2 expression through R‐loop formation and reinforced enhancer–promoter communication, thereby converting *Kdm5a*‐loss‐induced epigenetic activation into persistent transcriptional reinforcement. In mice with endothelial‐specific deletion of *Kdm5a*, this mechanism promotes the maturation and stabilization of nascent‐microvascular networks after myocardial infarction. In a clinical cohort, serum soluble TIE2 independently stratified collateral status in 402 patients with chronic total occlusion. To further enable translation, we developed LNP@Tek‐e1 modified with a VCAM‐1‐targeting peptide, combining VCAM‐1‐directed endothelial enrichment with enhancer‐dependent Tek‐e1 activity to selectively enhance *Tek*/TIE2 expression in border‐zone endothelium, improving microvascular function and limiting adverse left ventricular remodelling. Together, these findings define KDM5A downregulation as a hypoxia‐triggered endothelial repair programme and establish Tek‐e1 as an essential cis‐regulatory amplifier that can be delivered in vivo to restore post‐infarction microvascular homeostasis.

## Graphical abstract

Hypoxia‐induced KDM5A downregulation establishes a distinct endothelial repair programme, increasing H3K4me3 at the *Tek* promoter and intronic enhancer, enabling ISL1 to coordinately activate *Tek* transcription and induce enhancer‐RNA Tek‐e1. This cis‐regulatory RNA is essential for additional *Tek*/TIE2 induction through R‐loop formation and reinforced enhancer–promoter communication. VCAM‐1‐targeted LNP@Tek‐e1 further amplifies this response, restoring microvascular homeostasis and limiting adverse ventricular remodelling.

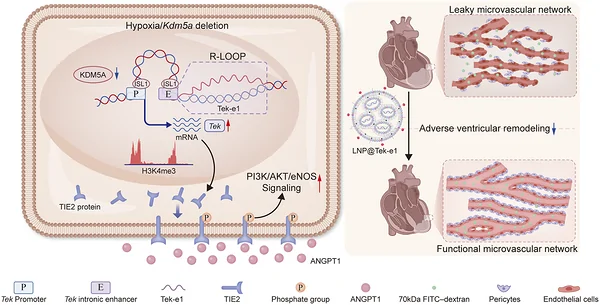

## 1 Introduction

Acute myocardial infarction (MI) remains a leading cause of chronic heart failure despite the widespread adoption of reperfusion therapy and contemporary antithrombotic treatment [1, 2]. Early restoration of myocardial perfusion is essential for minimizing infarct size and limiting cardiac damage [3]. Accordingly, promoting revascularization in the infarct zone has been pursued as an effective reparative strategy. MI triggers a rapid and robust angiogenic response; however, a purely quantitative increase in microvessels does not equate to effective perfusion [4]. Immature, unstabilized neovessels are prone to leakage, regression, and collapse, preventing the establishment of a durable microvascular network [5, 6]. By contrast, promoting microvascular homeostasis—characterized by reduced endothelial apoptosis, enlarged microvascular lumens, increased pericyte coverage, and enhanced barrier integrity—restores regional perfusion [5]. The endothelial homeostatic receptor TIE2 is a central regulator of this process, integrating angiopoietin signaling to stabilize nascent vessels and sustain microvascular perfusion [7]. Extensive clinical evidence indicates that indices of microvascular patency and integrity are independent predictors of major adverse cardiovascular events and left ventricular recovery after reperfusion [8, 9, 10, 11]. Therefore, focusing on post‐angiogenic homeostasis restoration is central to improving the quality of revascularization and long‐term prognosis.

Lysine‐specific demethylase 5A (KDM5A), a member of the KDM5/Jumonji family, removes di‐ and trimethyl groups from histone H3 lysine 4 (H3K4me2/3) and typically functions as a transcriptional corepressor [12]. KDM5A functions as both an enzymatic oxygen sensor and an epigenetic effector. Under hypoxic conditions, limited substrate availability diminishes KDM5A's demethylase activity, leading to promoter‐proximal accumulation of H3K4me3 at target genes [13]. In certain cell types, such as endometrial stromal cells, hypoxia also downregulates KDM5A expression, further altering H3K4me3 abundance and contributing to stress adaptation [14]. Additional evidence indicates that KDM5A tunes promoter H3K4me3 levels and transcriptional output to regulate angiogenic responses [15, 16, 17]. However, previous studies have largely focused on promoter‐proximal regulation and angiogenic growth. Whether hypoxia‐responsive KDM5A coordinates promoter and distal enhancer activity at the same endothelial locus to guide nascent neovessels toward stabilization and maturation remains unknown.

Enhancers are key cis‐regulatory elements that engage target gene promoters through three‐dimensional chromatin folding (looping) to cooperatively amplify transcription [18, 19]. Active enhancers are characterized by H3K4me1 along with H3K27ac, enrichment of p300 and RNA polymerase II (Pol II) at these regions, and the production of enhancer RNAs (eRNAs) [19, 20, 21]. Notably, H3K4me3 enrichment can also occur at enhancers, typically accompanied by increased enhancer activity and elevated eRNA transcription [22]. Activated enhancers promote transcription at target promoters by remodeling chromatin and organizing transcriptional complexes/condensates [23]. Prior studies have shown that eRNAs are closely associated with diverse diseases and can modulate gene expression through multiple mechanisms, including facilitating enhancer–promoter communication, stabilizing the transcriptional machinery, and regulating R‐loops. However, their precise functions remain under active investigation [19]. Growing evidence suggests that changes in enhancer function regulate multiple steps of revascularization in ischemic disease [16, 24]. Moreover, enhancers and their eRNAs exhibit strong cell‐type and context specificity, conferring inherent selectivity and targeting potential for enhancer‐ or eRNA‐based interventions [16].

Here, we define a hypoxia‐responsive endothelial repair programme in the infarct border zone and show how KDM5A‐loss‐induced chromatin remodelling primes the *Tek* locus, where the newly identified eRNA Tek‐e1 is required to reinforce enhancer‐promoter communication and restore microvascular homeostasis after MI.

## 2 Results

### 2.1 Border‐Zone Endothelial Cells Exhibit Enhanced Transcriptional Activity and KDM5A–H3K4me3 Remodeling

Vascular repair after myocardial infarction (MI) exhibits marked regional heterogeneity. To characterize the chromatin states of endothelial cells across different regions, we reanalyzed single‐nucleus assay for transposase‐accessible chromatin sequencing (snATAC‐seq) data from the remote zone (RZ), border zone (BZ), and infarct zone (IZ) of human infarcted hearts [25]. Reclustering of endothelial cells identified five clusters with distinct chromatin‐accessibility profiles. Among them, EC‐2 exhibited the highest gene‐associated chromatin‐accessibility score and was relatively enriched in the BZ (Figure 1A–D). Consistently, UMAP visualization and quantitative analysis showed that BZ endothelial cells had greater overall gene‐associated chromatin accessibility (Figure 1E). Because chromatin accessibility does not necessarily translate into actual transcriptional output, we next assessed nascent RNA synthesis using EU incorporation. At day 7 after MI, EU signals were significantly higher in BZ endothelial cells than in RZ endothelial cells (Figure 1F), indicating that BZ endothelial cells exhibit both increased chromatin accessibility and enhanced nascent transcriptional activity.

**FIGURE 1:**
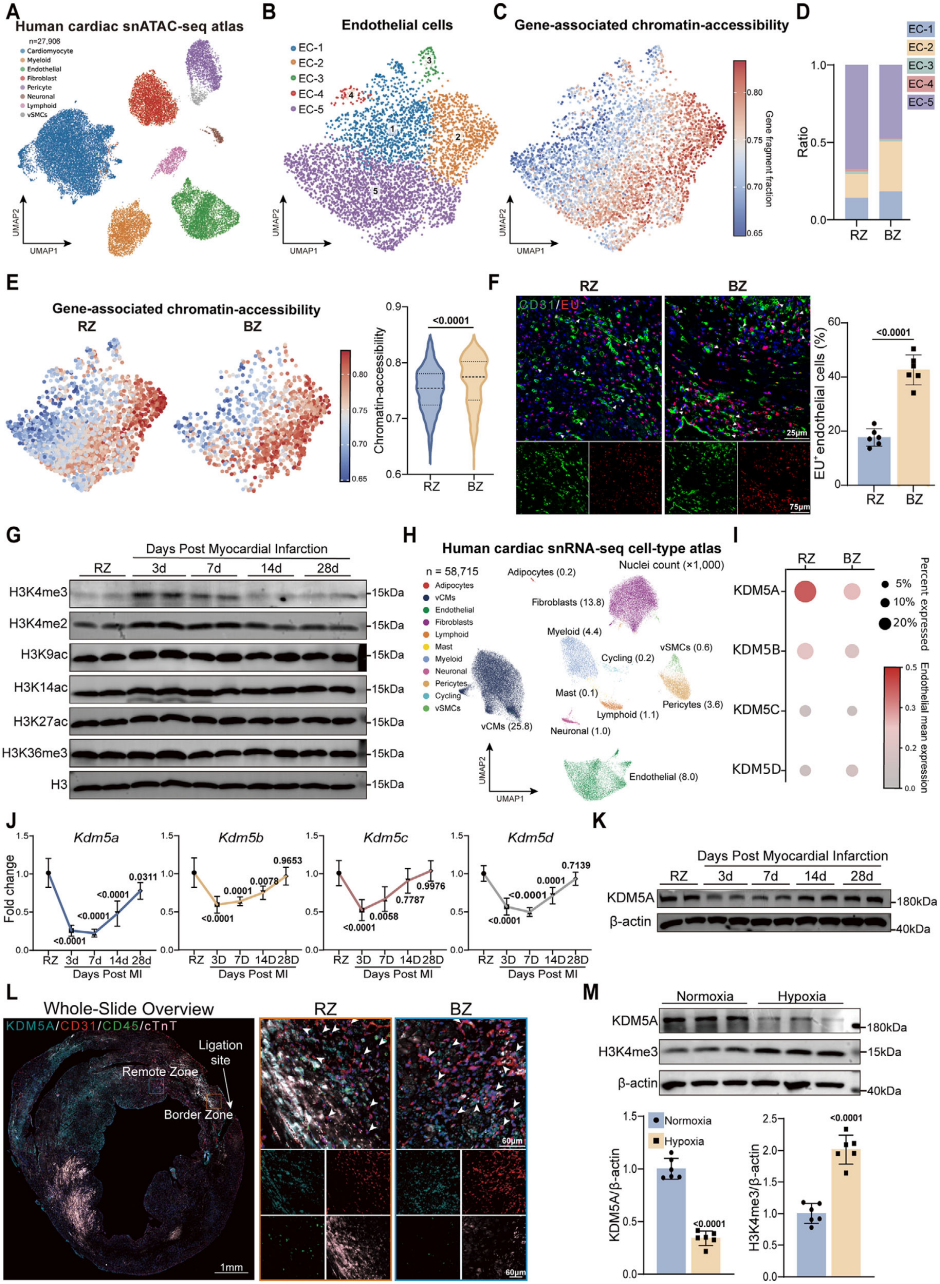
Border‐zone endothelial cells exhibit enhanced transcriptional activity and KDM5A–H3K4me3 remodeling. (A) UMAP of 27,906 nuclei from the human cardiac snATAC‐seq dataset, coloured by major cell type. (B) UMAP of endothelial nuclei resolved into five subclusters. (C) Gene‐associated chromatin accessibility across endothelial nuclei. (D) Relative abundance of EC‐1–EC‐5 in the RZ and BZ. (E) UMAP projections and quantification of gene‐associated chromatin accessibility in endothelial cells from the RZ and BZ (unpaired two‐tailed Student's *t*‐test). (F) Representative images and quantification of EU incorporation in CD31^+^ endothelial cells from the RZ and BZ at 7 days after MI (n  =  6 per group; unpaired two‐tailed Student's *t*‐test). (G) Representative immunoblots of the histone modifications in purified cardiac endothelial cells from RZ and the BZ at the indicated times after MI. (H) UMAP of 58,715 nuclei from the human cardiac snRNA‐seq cohort, coloured by cell type. (I) Dot plot of KDM5‐family expression in endothelial nuclei from the RZ and BZ. (J) RT–qPCR analysis of *Kdm5a–d* in purified mouse cardiac endothelial cells (n  =  6 per group; one‐way ANOVA with Tukey's multiple‐comparisons test). (K) Representative immunoblots of KDM5A in cardiac endothelial cells from RZ and the BZ at the indicated times after MI. (L) Whole‐section and enlarged multiplex TSA immunofluorescence images at 3 days after MI. (M) Representative immunoblots and quantification of KDM5A and H3K4me3 in mouse cardiac microvascular endothelial cells cultured under normoxia or hypoxia for 24 h (n  =  6 per group; unpaired two‐tailed Student's *t*‐test). RZ, remote zone; BZ, border zone. The data were presented as the means ± SD of at least three independent biological experiments.

To identify epigenetic alterations associated with this transcriptional feature, we compared cardiac endothelial cells isolated from the RZ with those isolated from the BZ at different time points after MI. The purity of the magnetically isolated cells was verified by flow cytometry (Figure S1A). Immunoblot screening of multiple histone methylation and acetylation marks revealed that H3K4me2 and H3K4me3 showed the most pronounced time‐dependent increases, whereas the other histone modifications examined did not display consistent changes (Figures 1G and S1B). These findings identify H3K4 methylation as a prominent feature of endothelial epigenetic remodeling after MI.

Given that H3K4me2/3 are primarily regulated by KDM5 family demethylases, we next examined the expression of individual KDM5 family members. Among purified cardiac endothelial cells, *Kdm5a* showed the most pronounced reduction during the early phase after MI, accompanied by a corresponding decrease in KDM5A protein abundance (Figures 1J,K and S1E). To assess whether this alteration was conserved in human MI, we reanalyzed a human cardiac snRNA‐seq dataset and constructed an atlas of the major cardiac cell types (Figures 1H and S1C). In human endothelial nuclei, KDM5A expression was relatively preserved in the RZ but was markedly reduced in the BZ. By contrast, the other KDM5 family members did not exhibit consistent region‐specific changes (Figure 1I). Moreover, KMT2 family members did not show coordinated upregulation in the BZ consistent with H3K4me2/3 accumulation (Figure S1D).

Although the isolated‐cell and single‐nucleus transcriptomic analyses both indicated reduced endothelial KDM5A expression, its spatial localization within the tissue and its selectivity relative to other major cardiac cell types remained to be defined. Multiplex TSA immunofluorescence showed that KDM5A reduction was predominantly localized to CD31^+^ endothelial cells in the BZ (Figure 1L). We further magnetically separated leukocytes, endothelial cells, and other cardiac cell fractions from hearts at day 3 after MI. *Kdm5a* mRNA and KDM5A protein showed the greatest reductions in endothelial cells, which also exhibited the most pronounced H3K4me3 accumulation (Figure S1F–K). Together, the in situ localization and cell‐fractionation analyses demonstrate that early KDM5A downregulation and H3K4me3 elevation after MI occur predominantly in endothelial cells within the BZ.

Finally, to investigate a potential upstream trigger of this epigenetic alteration, we exposed mouse cardiac microvascular endothelial cells to hypoxia. Hypoxia similarly induced KDM5A downregulation and H3K4me3 accumulation (Figure 1M). Collectively, BZ endothelial cells exhibit increased chromatin accessibility and enhanced nascent transcriptional activity after MI, with hypoxia‐induced KDM5A downregulation and H3K4me3 accumulation constituting a prominent epigenetic basis for this hypertranscriptional feature.

### 2.2 Endothelial *Kdm5a* Deletion Favors Microvascular Stabilization Over Angiogenic Initiation

To determine whether endothelial KDM5A downregulation is sufficient to recapitulate the transcriptional activation observed in the infarct border zone and to define its functional consequences, we generated endothelial‐specific *Kdm5a* conditional knockout mice (Cdh5‐Cre; *Kdm5a* ^fl/fl^, hereafter *Kdm5a* ^cKO^), with littermate *Cdh5* ^Cre^ mice serving as controls. Primary mouse cardiac microvascular endothelial cells were isolated and cultured, and efficient loss of KDM5A was confirmed by immunoblotting (Figure 2A). EU incorporation assays showed a significant increase in nuclear EU signals in *Kdm5a* ^cKO^ endothelial cells (Figure 2B), demonstrating that KDM5A loss is sufficient to enhance nascent RNA synthesis in endothelial cells.

**FIGURE 2:**
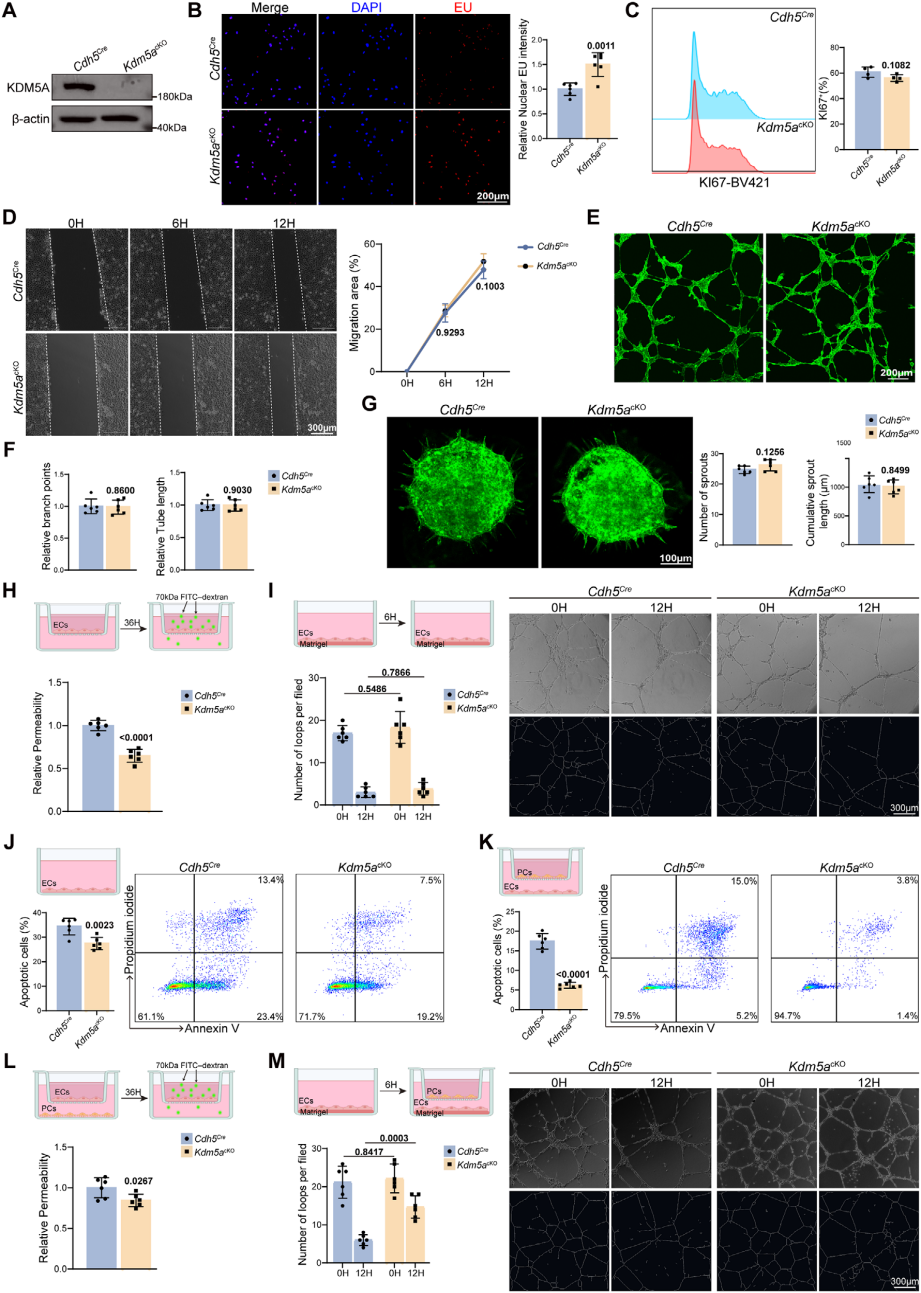
Endothelial *Kdm5a* deletion favors microvascular stabilization over angiogenic initiation. (A) Immunoblot analysis of KDM5A in primary cardiac microvascular endothelial cells isolated from *Cdh5* ^Cre^ and *Kdm5a* ^cKO^ mice. (B) EU incorporation and quantification of nuclear EU intensity in *Cdh5* ^Cre^ and *Kdm5a* ^cKO^ endothelial cells (n = 6 per group; unpaired two‐tailed Student's t‐test). (C) Flow‐cytometric analysis and quantification of Ki67^+^ *Cdh5* ^Cre^ and *Kdm5a* ^cKO^ endothelial cells (n = 6 per group; unpaired two‐tailed Student's t‐test). (D) Scratch‐wound migration assays in *Cdh5* ^Cre^ and *Kdm5a* ^cKO^ endothelial cells at 0, 6, and 12 h, with quantification of the migrated area (n = 6 per group; t unpaired two‐tailed Student's t‐test). (E, F) Matrigel tube formation by *Cdh5* ^Cre^ and *Kdm5a* ^cKO^ endothelial cells (E), with quantification of branch points and total tube length (F) (n = 6 per group; unpaired two‐tailed Student's t‐test). (G) Three‐dimensional spheroid sprouting of *Cdh5* ^Cre^ and *Kdm5a* ^cKO^ endothelial cells, with quantification of sprout number and cumulative sprout length (n = 6 per group; unpaired two‐tailed Student's t‐test). (H–J) Functional characterization of *Cdh5* ^Cre^ and *Kdm5a* ^cKO^ endothelial cells in monoculture. (H) Transendothelial permeability to 70‐kDa FITC–dextran measured 36 h after cell seeding (n = 6 per group; unpaired two‐tailed Student's t‐test). (I) Matrigel network regression at 0 and 12 h, with quantification of the number of loops per field. Skeletonized networks are shown in the lower panels (n = 6 per group; two‐way ANOVA with Tukey's multiple‐comparisons test). (J) Annexin V/propidium iodide flow‐cytometric analysis and quantification of endothelial apoptosis under hypoxia (n = 6 per group; unpaired two‐tailed Student's t‐test). (K–M) Functional characterization of *Cdh5* ^Cre^ and *Kdm5a* ^cKO^ endothelial cells cocultured with pericytes. (K) Annexin V/propidium iodide flow‐cytometric analysis and quantification of endothelial apoptosis under hypoxia (n = 6 per group; unpaired two‐tailed Student's t‐test). (L) Transendothelial permeability to 70‐kDa FITC–dextran measured 36 h after cell seeding (n = 6 per group; unpaired two‐tailed Student's t‐test). (M) Matrigel network regression at 0 and 12 h, with quantification of the number of loops per field. Skeletonized networks are shown in the lower panels (n = 6 per group; two‐way ANOVA with Tukey's multiple‐comparisons test). ECs, endothelial cells; PCs, pericytes. Data are presented as mean ± SD. Each dot represents an independent biological replicate. Created in BioRender. Yang, L. (2026) https://BioRender.com/3z9ruj1.

Post‐ischemic microvascular repair comprises the initial formation of vascular networks through endothelial proliferation, migration, and sprouting, followed by the maintenance of endothelial survival, barrier integrity, and network architecture. Subsequent acquisition of pericyte coverage allows these vessels to progressively stabilize into persistent, functional microvessels [26]. We therefore evaluated the effects of KDM5A loss on both angiogenic initiation and post‐formation network stability. KDM5A loss did not significantly alter the proportion of Ki67^+^ endothelial cells or endothelial migration (Figure 2C,D). In Matrigel tube‐formation assays, the number of branch points and total tube length were comparable between the two groups (Figure 2E,F). Similarly, three‐dimensional endothelial spheroid assays showed no significant differences in sprout number or cumulative sprout length (Figure 2G). Thus, the transcriptional enhancement induced by KDM5A loss does not promote endothelial proliferation, migration, or initial vascular network formation.

We next assessed barrier function, survival under hypoxia, and post‐formation network maintenance in endothelial monocultures. KDM5A loss significantly reduced the transendothelial permeability of 70‐kDa FITC–dextran and decreased hypoxia‐induced endothelial apoptosis (Figure 2H,J). However, preformed networks in both groups underwent substantial regression over time, with no significant difference in the number of residual networks at 12 h (Figure 2I).

Because the stability of newly formed microvessels depends on interactions between endothelial cells and pericytes, we further established an endothelial cell–pericyte coculture system. *Kdm5a* ^cKO^ endothelial cells exhibited further reductions in apoptosis under hypoxic conditions and also showed reduced transendothelial permeability (Figure 2K,L). The two groups formed comparable initial networks at 0 h, whereas significantly more intact networks were retained in the *Kdm5a* ^cKO^ group after 12 h (Figure 2M). These results indicate that pericyte support further augments the barrier‐protective and prosurvival effects of KDM5A loss, while enhancing the stability of established vascular networks.

Collectively, KDM5A downregulation defines an endothelial hypertranscriptional state with microvascular stabilization, rather than angiogenic initiation, as its predominant functional output and may represent an endogenous reparative program that drives microvascular homeostatic restoration in the infarct border zone.

### 2.3 KDM5A Restricts *Tek*/TIE2 Activation Through Focal H3K4me3 Remodeling at the *Tek* Promoter and Intronic Enhancer

At day 3 after myocardial infarction, we isolated CD45^−^ CD31^+^ endothelial cells from the border zone and performed H3K4me3 CUT&Tag sequencing. The relative genomic distribution of H3K4me3 peaks across functional regions was broadly comparable between *Cdh5* ^Cre^ and *Kdm5a* ^cKO^ endothelial cells (Figure 3A). However, peak‐centered metaprofiles and heatmap visualization revealed a pronounced increase in H3K4me3 enrichment at a subset of genomic loci following *Kdm5a* deletion (Figure 3B). Annotation of differentially enriched H3K4me3 peaks identified 123 associated genes, including 107 linked to increased and 16 linked to decreased H3K4me3 enrichment, whereas the vast majority of H3K4me3‐marked loci showed no significant change (Figure 3C–E). Thus, KDM5A regulation of endothelial H3K4me3 after MI primarily involves locus‐specific remodeling rather than widespread changes across the genome.

**FIGURE 3:**
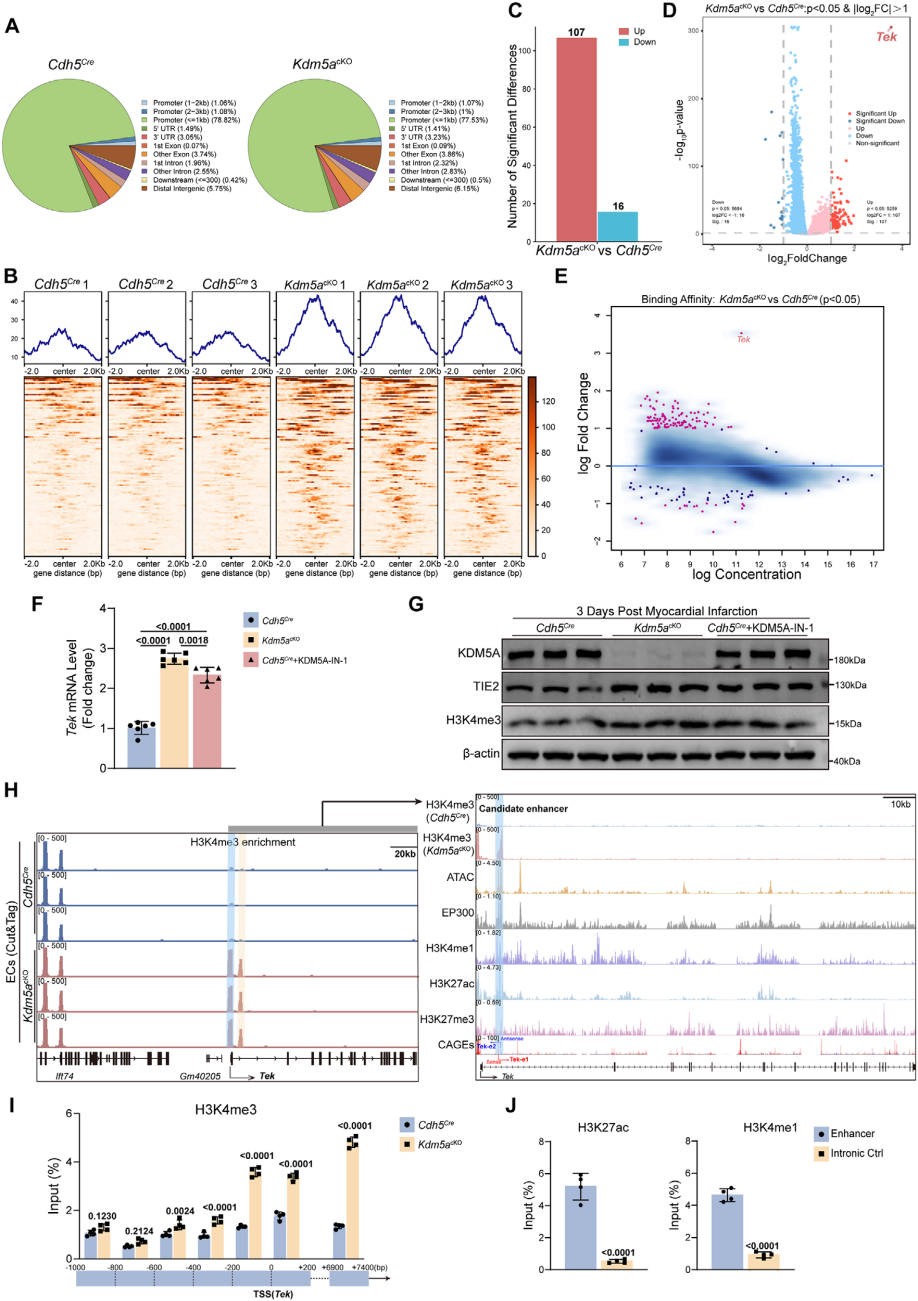
KDM5A restricts *Tek*/TIE2 activation through focal H3K4me3 remodeling at the *Tek* promoter and intronic enhancer. (A) Genomic annotation of H3K4me3 peaks in border‐zone cardiac endothelial cells isolated from *Cdh5* ^Cre^ and *Kdm5a* ^cKO^ mice at 3 days after MI. (B) Peak‐centered metaprofiles and heatmaps of H3K4me3 CUT&Tag enrichment within ±2 kb of peak centers in *Cdh5* ^Cre^ and *Kdm5a* ^cKO^ endothelial cells (n = 3 per group). (C) Numbers of genes associated with significantly increased or decreased H3K4me3 enrichment in *Kdm5a* ^cKO^ versus *Cdh5* ^Cre^ endothelial cells. (D, E) Volcano plot (D) and MA plot (E) of differential H3K4me3 enrichment in *Kdm5a* ^cKO^ versus *Cdh5* ^Cre^ endothelial cells. (F) RT–qPCR analysis of *Tek* mRNA in border‐zone endothelial cells isolated from *Cdh5* ^Cre^, *Kdm5a* ^cKO^, and KDM5A‐IN‐1‐treated *Cdh5* ^Cre^ mice at 3 days after MI (n = 6 per group; one‐way ANOVA with Tukey's multiple‐comparisons test). (G) Immunoblot analysis of KDM5A, TIE2, and H3K4me3 in border‐zone endothelial cells isolated from *Cdh5* ^Cre^, *Kdm5a* ^cKO^, and KDM5A‐IN‐1‐treated *Cdh5* ^Cre^ mice at 3 days after MI. (H) Genome‐browser tracks showing H3K4me3 CUT&Tag enrichment across the *Tek* locus in *Cdh5* ^Cre^ and *Kdm5a* ^cKO^ endothelial cells. The enlarged region shows publicly available ATAC‐seq, EP300, H3K4me1, H3K27ac, H3K27me3, and CAGE tracks at the candidate intronic enhancer. (I) H3K4me3 ChIP–qPCR across the *Tek* promoter and intronic enhancer in *Cdh5* ^Cre^ and *Kdm5a* ^cKO^ endothelial cells. Primer positions relative to the *Tek* transcription start site are indicated (n = 4 per group; unpaired two‐tailed Student's t‐test). (J) ChIP–qPCR analysis of H3K27ac and H3K4me1 enrichment at the *Tek* intronic enhancer and an intronic control region (n = 4 per group; unpaired two‐tailed Student's t‐test). BZ, border zone; MI, myocardial infarction; TSS, transcription start site. Data are presented as mean ± SD. Each dot represents an independent biological replicate.

*Tek* showed the most pronounced increase in H3K4me3 among the KDM5A‐sensitive genes. *Kdm5a* deletion markedly increased H3K4me3 deposition at the *Tek* locus and was accompanied by increased *Tek* transcription (Figure 3D–F). Because KDM5A has both catalytic and non‐catalytic functions, we next examined the contribution of its demethylase activity to this process. Following MI, Cdh5^Cre^ mice were treated with KDM5A‐IN‐1, and border‐zone endothelial cells were isolated on day 3. Pharmacological inhibition of KDM5A demethylase activity significantly increased *Tek* mRNA, H3K4me3, and TIE2 protein levels without reducing KDM5A protein abundance (Figures 3F,G and S2A). Together, the genetic and pharmacological findings support that KDM5A demethylase activity restricts H3K4me3 accumulation at the *Tek* locus and limits *Tek*/TIE2 expression.

To assess whether elevated TIE2 expression was accompanied by enhanced ligand responsiveness, we examined ANGPT1‐induced signaling in primary cardiac microvascular endothelial cells. Basal PI3K–AKT–eNOS pathway activity was low in both genotypes. Following ANGPT1 stimulation, *Kdm5a* ^cKO^ endothelial cells exhibited greater phosphorylation of PI3K, AKT, and eNOS, together with increased VE‐cadherin abundance (Figure S2B). These results indicate that *Kdm5a* deletion enhances endothelial responsiveness to ANGPT1.

To further define the cis‐regulatory regions within the *Tek* locus that respond to KDM5A loss, we systematically examined the distribution of H3K4me3 across this locus. Notably, in addition to the promoter‐associated peak near the transcription start site (TSS), a second KDM5A‐sensitive H3K4me3 peak was detected in the first intron of *Tek*, approximately 7 kb downstream of the TSS (Figure 3H). Integration of public epigenomic datasets showed that this region exhibited accessible chromatin, EP300 binding, enrichment of H3K4me1 and H3K27ac, reduced H3K27me3, and bidirectional CAGE signals, consistent with the features of an active intronic enhancer [27, 28, 29, 30, 31, 32, 33]. ChIP–qPCR further confirmed that this candidate region was significantly enriched for H3K27ac and H3K4me1 relative to a control region within the same intron (Figure 3J).

Consistent with the CUT&Tag results, H3K4me3 ChIP–qPCR showed that *Kdm5a* deletion increased H3K4me3 enrichment at both the *Tek* intronic enhancer and promoter, with the most pronounced change observed within the −200‐ to +200‐bp region surrounding the TSS (Figure 3I). These findings extend the regulatory scope of KDM5A at the *Tek* locus from the promoter to an intronic enhancer and indicate that KDM5A demethylase activity constrains *Tek*/TIE2 expression by limiting local H3K4me3 accumulation at both cis‐regulatory elements.

### 2.4 KDM5A Loss Enables ISL1‐Dependent Coactivation of *Tek* and the Enhancer RNA Tek‐e1

*Kdm5a* deletion induced concordant H3K4me3 enrichment at the *Tek* promoter and enhancer, suggesting coordinated regulation of the two cis‐regulatory elements. Because enhancer transcription often accompanies target‐gene activation, the prominent bidirectional CAGE signals at this region prompted us to examine enhancer‐derived transcripts. Integration of the transcriptional orientations defined by CAGE with 3′ RACE identified two previously unannotated enhancer‐derived transcripts, which we designated Tek‐e1 and Tek‐e2. Tek‐e1 was transcribed in the same orientation as *Tek*, whereas Tek‐e2 was transcribed in the opposite direction (Figure 4A). In primary cardiac microvascular endothelial cells, Tek‐e1 was significantly more abundant than Tek‐e2 and shared the same transcriptional orientation as *Tek*; Tek‐e1 was therefore prioritized for subsequent investigation (Figure 4B). Absolute quantification across multiple tissues, normalized to *Tek* pre‐mRNA, showed that Tek‐e1 was preferentially expressed in the heart and lung, indicating marked tissue selectivity (Figure 4C). Tek‐e1 expression was also significantly increased in *Kdm5a* ^cKO^ endothelial cells (Figure 4D). These findings identify Tek‐e1 as a previously unannotated, KDM5A‐responsive enhancer RNA with preferential cardiac expression.

**FIGURE 4:**
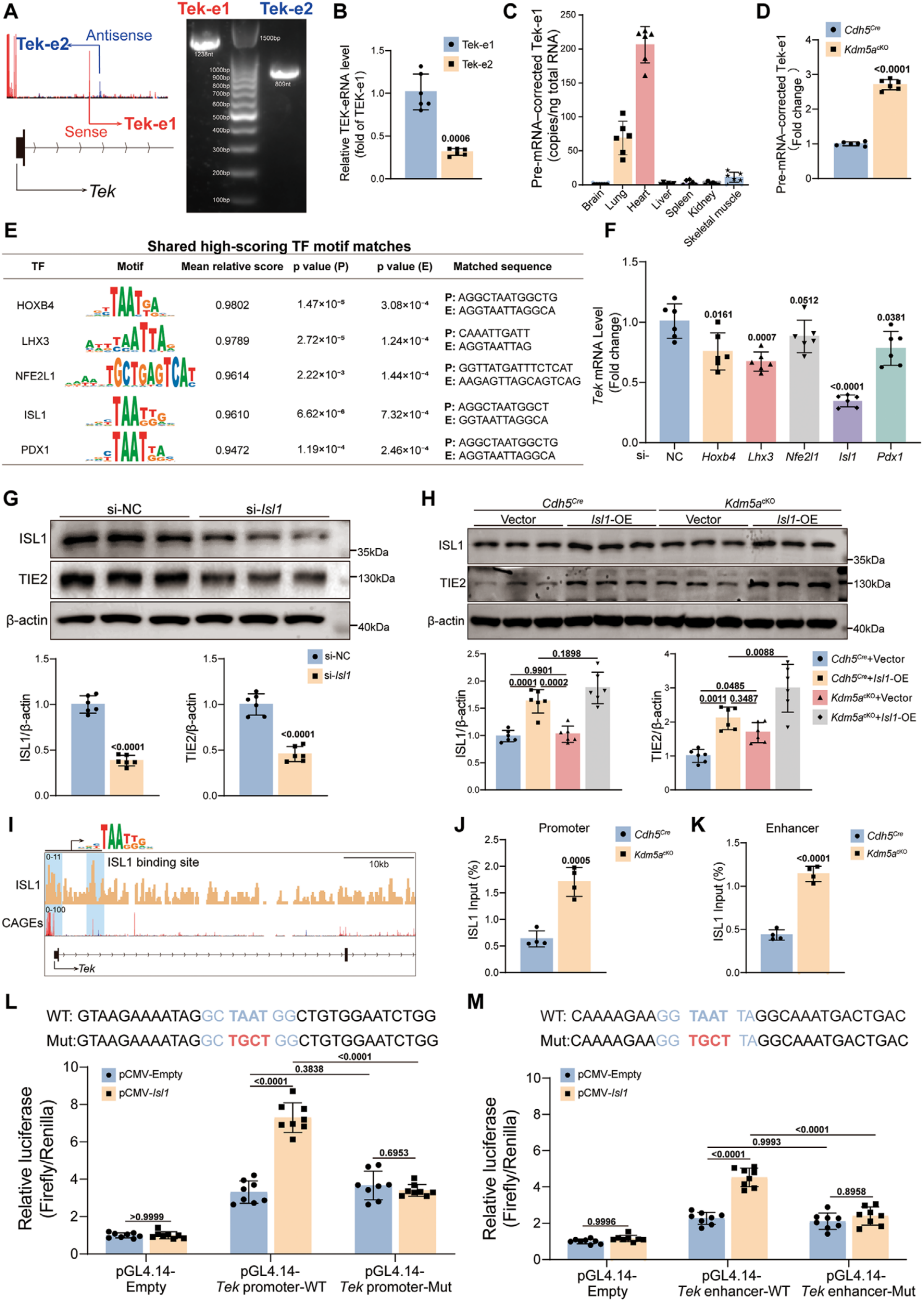
KDM5A loss enables ISL1‐dependent coactivation of *Tek* and the enhancer RNA Tek‐e1. (A) Bidirectional CAGE signals at the *Tek* enhancer and agarose‐gel analysis of 3′ RACE products corresponding to the previously unannotated transcripts Tek‐e1 and Tek‐e2. (B) RT–qPCR analysis of Tek‐e1 and Tek‐e2 abundance in primary cardiac microvascular endothelial cells (n = 6 per group; paired two‐tailed Student's t‐test). (C) Absolute quantification of Tek‐e1 across the indicated mouse tissues after normalization to *Tek* pre‐mRNA (n = 6 per tissue). (D) RT–qPCR analysis of Tek‐e1 in *Cdh5* ^Cre^ and *Kdm5a* ^cKO^ endothelial cells (n = 6 per group; unpaired two‐tailed Student's t‐test). (E) Spatzie‐based paired motif analysis of the *Tek* core promoter and the enhancer region surrounding the CAGE‐defined Tek‐e1 transcription start region. (F) siRNA screening of candidate transcription factors, with *Tek* mRNA quantified by RT–qPCR after silencing of Hoxb4, Lhx3, Nfe2l1, Isl1, or Pdx1 (n = 6 per group; one‐way ANOVA with Tukey's multiple comparisons test). (G) Immunoblot analysis and quantification of ISL1 and TIE2 in endothelial cells transfected with NC or *Isl1*‐targeting siRNA (n = 6 per group; unpaired two‐tailed Student's t‐test). (H) Immunoblot analysis and quantification of ISL1 and TIE2 in *Cdh5* ^Cre^ and *Kdm5a* ^cKO^ endothelial cells transfected with empty vector or an ISL1‐expression construct (n = 6 per group; two‐way ANOVA with Tukey's multiple‐comparisons test). (I) Genome‐browser tracks showing ISL1 ChIP–seq and CAGE signals across the *Tek* locus. (J, K) ISL1 ChIP–qPCR at the *Tek* promoter (J) and enhancer (K) in *Cdh5* ^Cre^ and *Kdm5a* ^cKO^ endothelial cells (n = 4 per group; unpaired two‐tailed Student's t‐test). (L) Dual‐luciferase analysis of wild‐type and ISL1‐motif‐mutant *Tek* promoter reporters following transfection with empty vector or an ISL1‐expression construct (n = 8 per group; two‐way ANOVA with Tukey's multiple‐comparisons test). (M) Dual‐luciferase analysis of wild‐type and ISL1‐motif‐mutant *Tek* enhancer reporters following transfection with empty vector or an ISL1‐expression construct (n = 8 per group; two‐way ANOVA with Tukey's multiple‐comparisons test). P, promoter; E, enhancer; NC, negative control; OE, overexpression. Data are presented as mean ± SD. Each dot represents an independent biological replicate.

Following *Kdm5a* deletion, concordant H3K4me3 enrichment at the *Tek* promoter and enhancer was accompanied by increased transcription of *Tek* and Tek‐e1, respectively, suggesting that shared sequence‐specific transcription factors might coordinate transcriptional output from the two elements. We therefore applied the Spatzie computational framework to perform paired motif analysis of the *Tek* promoter and the enhancer region centered on the CAGE‐defined Tek‐e1 transcription initiation region. This analysis prioritized HOXB4, LHX3, NFE2L1, ISL1, and PDX1 as candidate transcription factors with high‐scoring motifs shared between the two regions (Figure 4E). Among these candidates, *Isl1* silencing produced the most pronounced reduction in *Tek* mRNA (Figures 4F and S3A) and significantly decreased TIE2 protein abundance (Figure 4G). Conversely, ISL1 overexpression increased TIE2 expression and further enhanced TIE2 abundance in the context of *Kdm5a* deletion (Figure 4H).

Consistent with the motif predictions, ISL1 ChIP–seq revealed binding at both the *Tek* promoter and the Tek‐e1 transcription initiation region (Figure 4I). ChIP–qPCR confirmed ISL1 occupancy at both sites and showed that *Kdm5a* deletion significantly increased ISL1 enrichment at the promoter and Tek‐e1 transcription initiation region (Figure 4J,K). Meanwhile, H3K4me3 CUT&Tag and ChIP–qPCR analyses showed that *Kdm5a* deletion did not significantly alter H3K4me3 enrichment at the *Isl1* locus (Figure S3B,C). Similarly, *Isl1* mRNA and protein expression levels, as well as the nuclear‐to‐cytoplasmic distribution of ISL1, remained unchanged (Figure 4H and S3D,E). To determine whether ISL1‐mediated activation of these elements required the predicted binding sequences, we generated wild‐type reporter constructs containing either the *Tek* promoter or an enhancer fragment encompassing the Tek‐e1 transcription initiation region and introduced mutations into the corresponding core ISL1 motifs. ISL1 overexpression markedly increased the activity of both wild‐type reporters, whereas mutation of the ISL1‐binding motifs largely abolished both responses (Figure 4L,M).

Collectively, *Kdm5a* deletion establishes a permissive, H3K4me3‐enriched chromatin environment at the *Tek* promoter and enhancer and increases ISL1 occupancy at both cis‐regulatory elements, thereby enabling motif‐dependent coactivation of *Tek* and the newly identified enhancer RNA Tek‐e1.

### 2.5 Tek‐e1 is Required to Sustain *Tek*/TIE2 Expression Through R‐Loop‐Dependent Enhancer–Promoter Communication

To determine whether Tek‐e1 is merely a by‐product of enhancer activation or a functional RNA required for *Tek* transcription, we deleted a 20‐bp sequence centered on the Tek‐e1 transcription initiation region in mouse cardiac microvascular endothelial cells, generating Tek‐e1‐deficient cells. Tek‐e1 deficiency significantly reduced basal *Tek* mRNA levels. ISL1 overexpression markedly increased *Tek* expression in control cells, whereas this additional induction was completely abolished in Tek‐e1‐deficient cells (Figure 5A). Thus, Tek‐e1 contributes to basal *Tek* transcription and is required for further ISL1‐dependent activation.

**FIGURE 5:**
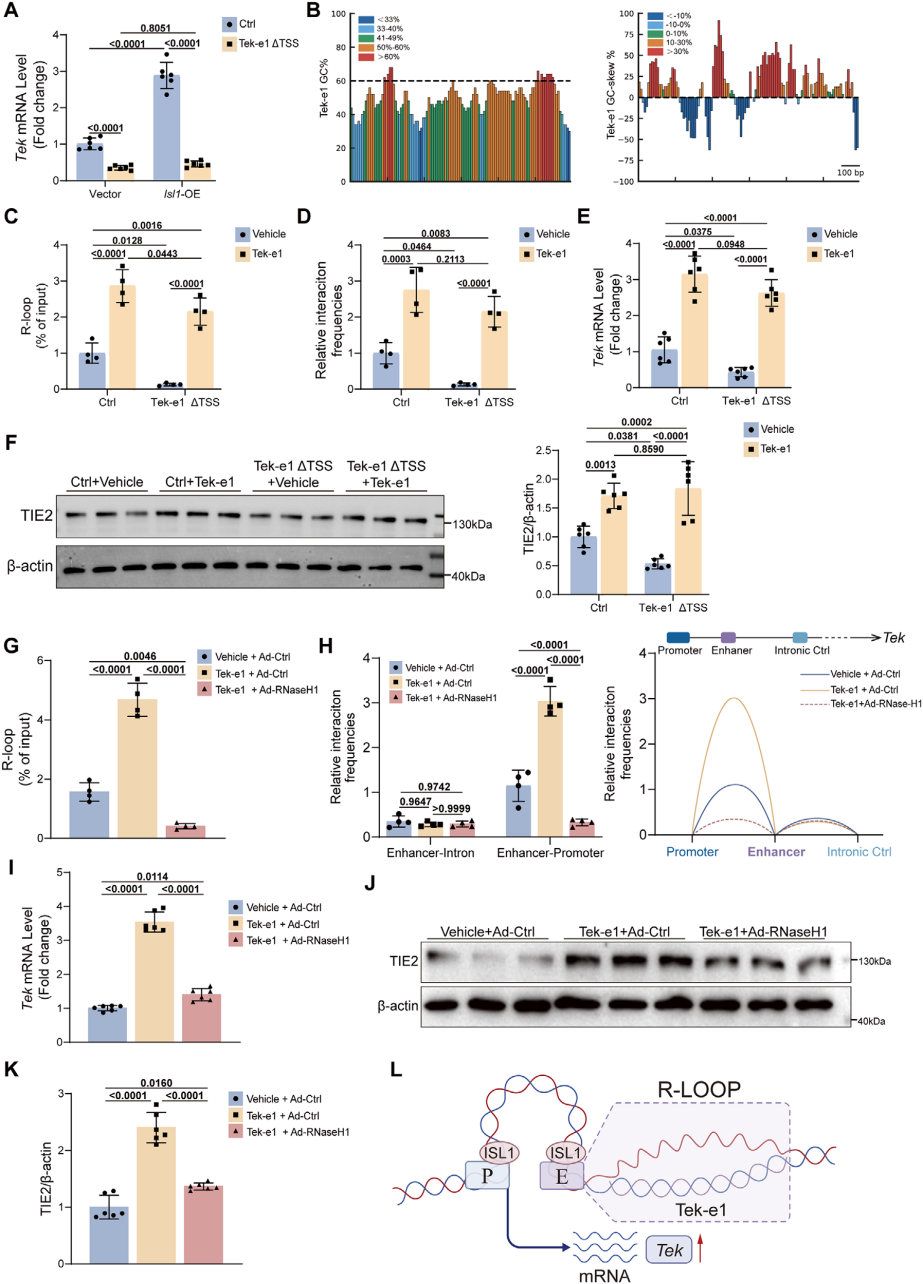
Tek‐e1 is required to sustain *Tek*/TIE2 expression through R‐loop‐dependent enhancer–promoter communication. (A) RT–qPCR analysis of *Tek* mRNA in control and Tek‐e1 ΔTSS endothelial cells transfected with empty vector or an ISL1‐expression construct. Tek‐e1 ΔTSS denotes cells carrying a 20‐bp deletion centered on the Tek‐e1 transcription initiation region (n = 6 per group; two‐way ANOVA with Tukey's multiple‐comparisons test). (B) Sliding‐window analysis of GC content and GC skew across the Tek‐e1 sequence. (C–F) Rescue of Tek‐e1 function in control and Tek‐e1 ΔTSS endothelial cells following delivery of Tek‐e1 carrying mouse SIRLOIN nuclear‐ enrichment elements. (C) DRIP–qPCR analysis of DNA:RNA hybrids at the Tek‐e1‐producing enhancer (n = 4 per group; two‐way ANOVA with Tukey's multiple‐comparisons test). (D) 3C–qPCR analysis of contact frequency between the Tek‐e1‐producing enhancer and the promoter (n = 4 per group; two‐way ANOVA with Tukey's multiple‐comparisons test). (E) RT–qPCR analysis of *Tek* mRNA (n = 6 per group; two‐way ANOVA with Tukey's multiple‐comparisons test). (F) Immunoblot analysis and quantification of TIE2 protein (n = 6 per group; two‐way ANOVA with Tukey's multiple‐comparisons test). (G–K) Analysis of the R‐loop dependence of Tek‐e1‐mediated transcriptional regulation in endothelial cells treated with vehicle plus control adenovirus, Tek‐e1 plus control adenovirus, or Tek‐e1 plus RNase H1‐expressing adenovirus. (G) DRIP–qPCR analysis of DNA:RNA hybrids at the Tek‐e1‐producing enhancer (n = 4 per group; one‐way ANOVA with Tukey's multiple‐comparisons test). (H) 3C–qPCR analysis of interaction frequencies between the enhancer and either promoter or an intronic control region (n = 4 per group; two‐way ANOVA with Tukey's multiple‐comparisons test). (I) RT–qPCR analysis of *Tek* mRNA (n = 6 per group; one‐way ANOVA with Tukey's multiple‐comparisons test). (J, K) Immunoblot analysis (J) and quantification (K) of TIE2 protein (n = 6 per group; one‐way ANOVA with Tukey's multiple‐comparisons test). (L) Schematic model of the KDM5A–ISL1–Tek‐e1 regulatory axis. Ctrl, control; ΔTSS, deletion of the Tek‐e1 transcription initiation region; OE, overexpression; Ad‐Ctrl, control adenovirus; Ad‐RNase H1, RNase H1‐expressing adenovirus; DRIP, DNA:RNA immunoprecipitation; 3C, chromosome conformation capture. Data are presented as mean ± SD. Each dot represents an independent biological replicate.

We next examined the sequence features underlying the regulatory activity of Tek‐e1. Tek‐e1 contained multiple consecutive high‐GC windows (>60%), clustered positive GC‐skew segments, and pronounced strand asymmetry, whereas these features were markedly attenuated in Tek‐e2 (Figures 5B and S4A). This sequence architecture confers a high potential for DNA:RNA hybridization, suggesting an intrinsic propensity of Tek‐e1 to form an R‐loop at its cognate enhancer.

Because deletion of the transcription initiation region also altered the local enhancer DNA sequence, we performed RNA rescue experiments to assess the function of the Tek‐e1 transcript. Delivery of in vitro‐transcribed Tek‐e1 carrying three mouse SIRLOIN nuclear‐ enrichment elements increased DNA:RNA hybrid signals at the cognate enhancer and enhanced contact between the enhancer and the *Tek* promoter (Figure 5C,D). In Tek‐e1‐deficient cells, exogenous Tek‐e1 also restored *Tek* mRNA and TIE2 protein levels (Figure 5E,F). These rescue results indicate that the local edit did not abolish the overall regulatory competence of the enhancer and that Tek‐e1 RNA can restore local DNA:RNA hybrid formation, enhancer–promoter communication, and *Tek*/TIE2 expression. These findings establish Tek‐e1 as a functional eRNA required for efficient *Tek* transcription rather than a passive by‐product of enhancer activation.

These results suggested that Tek‐e1 might promote enhancer–promoter communication through DNA:RNA hybrid formation but did not establish whether the resulting R‐loop was mechanistically required. To address this, we coexpressed a catalytically active, nucleus‐targeted RNase H1 during Tek‐e1 delivery to selectively resolve DNA:RNA hybrids. RNase H1 abolished the Tek‐e1‐induced hybrid signal at the enhancer (Figure 5G) and selectively reduced enhancer–*Tek* promoter contact, while exerting little effect on interactions between the enhancer and an intronic control region (Figure 5H). RNase H1 concomitantly reversed the Tek‐e1‐induced increases in *Tek* mRNA and TIE2 protein abundance (Figure 5I–K).

Tek‐e1‐induced R‐loop formation exhibited locus specificity. DRIP–qPCR analysis showed that Tek‐e1 supplementation did not significantly increase R‐loop enrichment at a *Tek* intronic control region or at two distal loci identified by BLAST based on sequence similarity to Tek‐e1 (Figure S4B). Consistently, S9.6 dot‐blot and immunoblot analyses revealed no significant changes in global DNA hybrid abundance or γH2AX levels (Figure S4C, D). These findings suggest that Tek‐e1 preferentially promotes R‐loop formation at its cognate enhancer without detectable global R‐loop accumulation or increased DNA damage markers.

Cross‐species comparative genomic analysis revealed sequence conservation of Tek‐e1 and its enhancer. Ensembl Compara LASTZ_NET alignments identified a region within the first intron of human *TEK* that exhibits substantial sequence homology to mouse Tek‐e1 (Figure S4E). This region harbors enhancer elements annotated by Ensembl and ENCODE, exhibits bidirectional transcription initiation signals detected by FANTOM5 CAGE, and displays pronounced H3K27ac and H3K4me3 enrichment in primary human endothelial cells (Figure S4F). These convergent lines of evidence demonstrate substantial sequence conservation of Tek‐e1 and identify a homologous, active enhancer with evidence of eRNA transcription at the corresponding human *TEK* locus.

Collectively, KDM5A downregulation establishes an H3K4me3‐enriched chromatin environment at the *Tek* promoter and enhancer, increasing ISL1 occupancy and coordinately inducing *Tek* and Tek‐e1 transcription. Tek‐e1 is subsequently required for ISL1‐dependent induction of endogenous *Tek* and acts through an RNase H1‐sensitive R‐loop to sustain enhancer–promoter communication, thereby supporting high‐level *Tek* transcription and TIE2 expression (Figure 5L).

### 2.6 Endothelial *Kdm5a* Deletion Restores Microvascular Homeostasis After Myocardial Infarction

The preceding findings showed that, under ischemic conditions, KDM5A downregulation was associated with increased *Tek*/TIE2 expression and a stabilization‐oriented endothelial phenotype. We therefore examined the in vivo relevance of this program during post‐MI repair. At 12 h after myocardial infarction, TTC staining showed no significant difference in the initial infarct area between *Kdm5a* ^cKO^ and *Cdh5* ^Cre^ mice (Figure S5A). Echocardiography revealed comparable left ventricular function between the two groups at baseline and on day 3 after MI. From day 7 onward, however, *Kdm5a* ^cKO^ mice exhibited persistently higher ejection fraction and fractional shortening, together with attenuated late left ventricular dilation (Figures 6A,B and S5B–D). By day 28, infarct scar size and collagen volume fraction were both significantly reduced in *Kdm5a* ^cKO^ hearts (Figure 6C).

**FIGURE 6:**
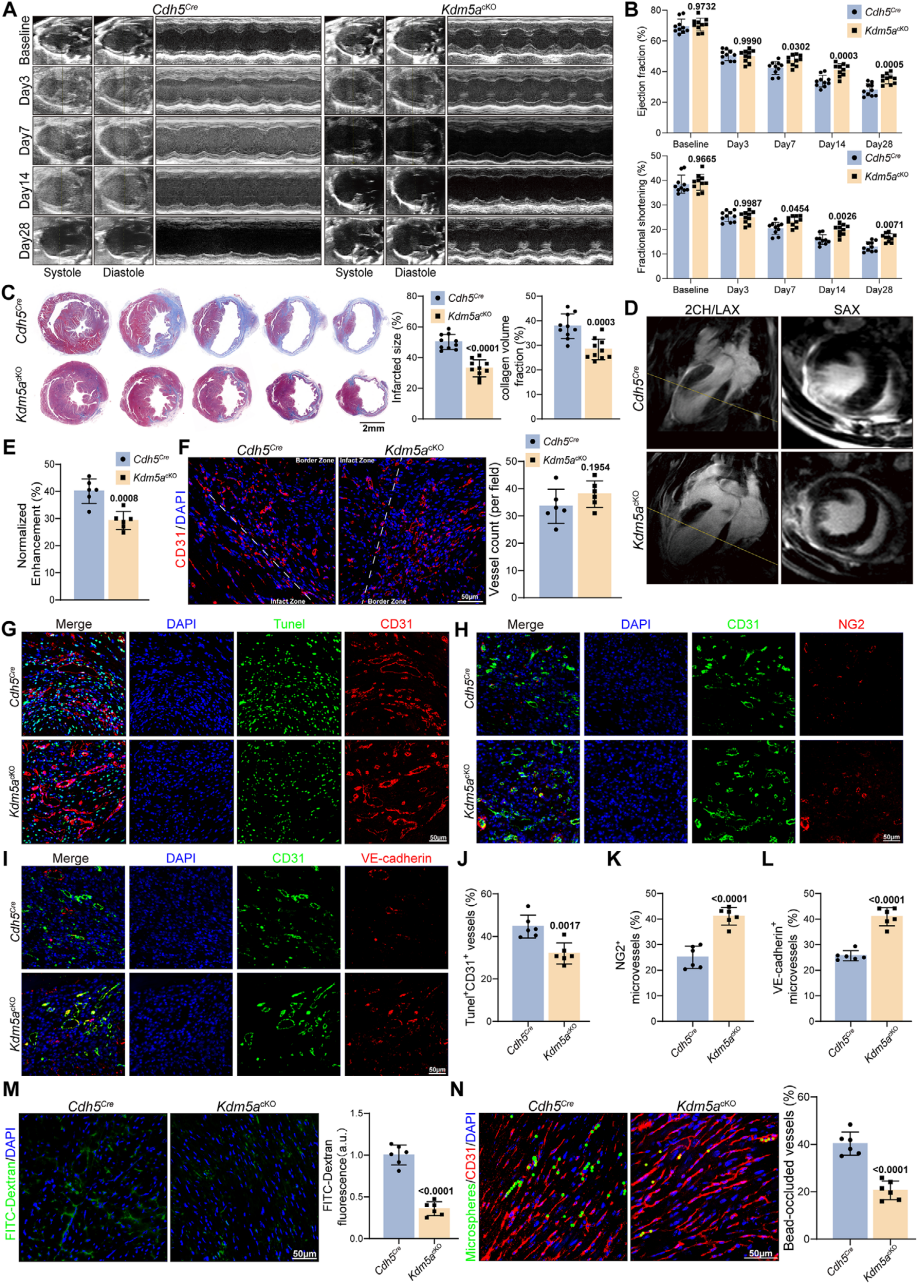
Endothelial *Kdm5a* deletion restores microvascular homeostasis after myocardial infarction. (A) M‐mode echocardiography of *Cdh5* ^Cre^ and *Kdm5a* ^cKO^ mice at baseline and on days 3, 7, 14, and 28 after MI. (B) Quantification of left ventricular ejection fraction and fractional shortening at the indicated time points (n = 10 mice per group; two‐way ANOVA with Sidak's multiple‐comparisons test). (C) Masson's trichrome staining of serial short‐axis heart sections and quantification of infarct size and collagen volume fraction on day 28 after MI (n = 10 mice per group; unpaired two‐tailed Student's t‐test). (D, E) Late gadolinium enhancement magnetic resonance imaging in two‐chamber long‐axis and short‐axis views (D) and quantification of normalized enhancement in the infarct border zone (E) on day 7 after MI (n = 6 mice per group; unpaired two‐tailed Student's t‐test). (F) CD31 immunofluorescence and quantification of microvessel number in the infarct border zone on day 3 after MI (n = 6 mice per group; unpaired two‐tailed Student's t‐test). (G–L) Analysis of microvascular stability in the infarct border zone on day 7 after MI. TUNEL and CD31 staining (G) and quantification of TUNEL^+^CD31^+^ vessels (J); CD31 and NG2 staining (H) and quantification of NG2^+^ pericyte‐covered microvessels (K); and CD31 and VE‐cadherin staining (I) and quantification of VE‐cadherin^+^ microvessels (L) (n = 6 mice per group; unpaired two‐tailed Student's t‐test). (M) Analysis of 70‐kDa FITC–dextran extravasation in the infarct border zone on day 7 after MI (n = 6 mice per group; unpaired two‐tailed Student's t‐test). (N) Analysis of microvessels occluded by 4‐µm FITC‐labeled carboxylated microspheres in the infarct border zone on day 7 after MI (n = 6 mice per group; unpaired two‐tailed Student's t‐test). MI, myocardial infarction; LGE‐MRI, late gadolinium enhancement magnetic resonance imaging; 2CH/LAX, two‐chamber long‐axis view; SAX, short‐axis view. Data are presented as mean ± SD. Each dot represents an independent mouse.

On day 7 after MI, late gadolinium enhancement magnetic resonance imaging showed reduced enhancement in the border zone of *Kdm5a* ^cKO^ hearts, suggesting enhanced local microvascular perfusion (Figure 6D,E). Because the preceding in vitro experiments showed no increase in endothelial proliferation or angiogenic activity following *Kdm5a* deletion, we next assessed endothelial proliferation and microvessel abundance in vivo. On day 3 after MI, Ki67 signal intensity in CD45^−^CD31^+^ endothelial cells was comparable between the two groups, and CD31 staining revealed no significant difference in border‐zone microvessel abundance (Figures S5E and 6F). Thus, the microcirculatory and cardiac benefits of *Kdm5a* deletion were not accompanied by enhanced angiogenesis.

We next examined microvascular stability and maturation in the border zone. On day 7 after MI, the proportion of TUNEL^+^CD31^+^ vessels was reduced in *Kdm5a* ^cKO^ hearts, whereas NG2^+^ pericyte coverage and VE‐cadherin^+^ endothelial junctional integrity were both significantly increased (Figure 6G–L). Consistent with these structural changes, 70‐kDa FITC–dextran tracing showed reduced vascular leakage in the border zone (Figure 6M). The proportion of microvessels occluded by 4‐µm FITC‐labeled carboxylated microspheres was also markedly reduced, indicating improved luminal patency and microvascular flow (Figure 6N).

Collectively, endothelial *Kdm5a* deletion promoted the survival, maturation, and functional stability of border‐zone microvessels without enhancing angiogenesis and was accompanied by sustained improvement in cardiac function and attenuation of adverse ventricular remodeling. These findings identify microvascular homeostatic restoration as the principal in vivo vascular phenotype associated with endothelial *Kdm5a* loss.

### 2.7 TIE2 Signaling is Required for *Kdm5a* loss–Induced Microvascular Homeostatic Restoration

The preceding findings showed that endothelial *Kdm5a* deletion enhances *Tek*/TIE2 expression through the H3K4me3–ISL1–Tek‐e1 regulatory axis and promotes microvascular homeostatic restoration in the infarct border zone. To determine whether TIE2 activity is required for this in vivo phenotype, we treated *Kdm5a* ^cKO^ mice with the TIE2 kinase inhibitor Compound 5. Compound 5 did not reduce the elevated TIE2 protein abundance in *Kdm5a* ^cKO^ endothelial cells but markedly suppressed PI3K, AKT, and eNOS phosphorylation and decreased VE‐cadherin expression (Figure S6A, B), indicating effective blockade of the downstream signaling output generated by increased TIE2 expression.

Compound 5 substantially attenuated the microvascular stabilization induced by *Kdm5a* deletion. In the infarct border zone, the proportions of microvessels covered by NG2^+^ pericytes and those with intact VE‐cadherin^+^ endothelial junctions were reduced (Figure S6C–F), accompanied by increased extravasation of 70‐kDa FITC–dextran and reduced microvascular luminal diameter (Figure S6G–J). LGE‐MRI further showed that Compound 5 restored the elevated late enhancement in the border zone of *Kdm5a* ^cKO^ hearts, suggesting loss of the local microvascular perfusion benefit (Figure S7A). These findings indicate that TIE2 activity is required for *Kdm5a* deletion to maintain microvascular maturation, stability, and luminal patency.

Loss of the microvascular homeostatic benefit further impaired myocardial repair. Echocardiography showed that Compound 5 markedly blunted the improvements in ejection fraction and fractional shortening observed in *Kdm5a* ^cKO^ mice on days 7 and 28 after MI and aggravated late left ventricular dilation (Figure S7B, C). By day 28, Compound 5 also significantly increased infarct scar size and collagen volume fraction in *Kdm5a* ^cKO^ hearts (Figure S7D).

The TIE2 agonist COMP‐Ang1 also exerted significant microvascular stabilizing effects in wild‐type mice following MI. COMP‐Ang1 treatment enhanced PI3K, AKT, and eNOS phosphorylation and increased VE‐cadherin expression (Figure S8A). Consistently, the proportions of NG2^+^ pericyte‐covered microvessels and VE‐cadherin^+^ microvessels were significantly increased in the infarct border zone (Figure S8B, C), accompanied by reduced FITC–dextran leakage and increased microvascular luminal diameter (Figure S8D, E). However, coadministration of Compound 5 markedly attenuated COMP‐Ang1‐induced downstream signaling activation and microvascular stability, indicating that the effects of COMP‐Ang1 depend on TIE2 kinase activity.

Collectively, pharmacological inhibition of TIE2 suppressed PI3K–AKT–eNOS signaling and substantially diminished the microvascular homeostatic and cardioprotective benefits conferred by endothelial *Kdm5a* deletion. Conversely, TIE2 activation by COMP‐Ang1 promoted microvascular maturation and functional stability, effects that were attenuated by TIE2 kinase inhibition. These findings further establish TIE2 as a critical effector linking KDM5A loss‐induced epigenetic and transcriptional remodeling to microvascular homeostatic restoration and cardiac protection.

### 2.8 A Clinically Relevant TIE2 Axis Supports Dual‐Selective Tek‐e1 Therapy for Ischemic Cardiac Repair

To assess the clinical relevance of the TIE2 axis in human ischemic vascular adaptation, we measured serum soluble TIE2 (sTIE2) levels in patients with coronary chronic total occlusion (CTO). Between February 2019 and September 2023, 493 consecutive patients were screened; after exclusion of 91 patients, 402 were included in the final analysis (Figure S9A) [3]. Of these, 23, 127, 140, and 112 patients had Rentrop grades 0, 1, 2, and 3, respectively. Patients were further classified as having poor collateral circulation (Rentrop grades 0–1) or good collateral circulation (Rentrop grades 2–3; Table S1).

Serum sTIE2 levels increased progressively with Rentrop grade (Figure 7A). Patients with good collateral circulation had significantly higher sTIE2 levels than those with poor collateral circulation [2413.50 (1835.75–2990.50) versus 1962.50 (1693.50–2331.25) pg/mL, P < 0.001], and sTIE2 correlated positively with Rentrop score (Spearman r = 0.270, P < 0.001). After dichotomization at the median value of 2163.75 pg/mL, unadjusted logistic regression showed that higher sTIE2 levels were significantly associated with good collateral circulation (OR = 2.990, 95% CI 1.968–4.588, P < 0.001). This association remained significant after adjustment for age and sex in model 1 (OR = 2.999, 95% CI 1.972–4.605, P < 0.001) and was not attenuated after further adjustment for body mass index, smoking, hypertension, diabetes, prior myocardial infarction, and serum creatinine in model 2 (OR = 3.325, 95% CI 2.112–5.315, P < 0.001; Table 1).

**FIGURE 7:**
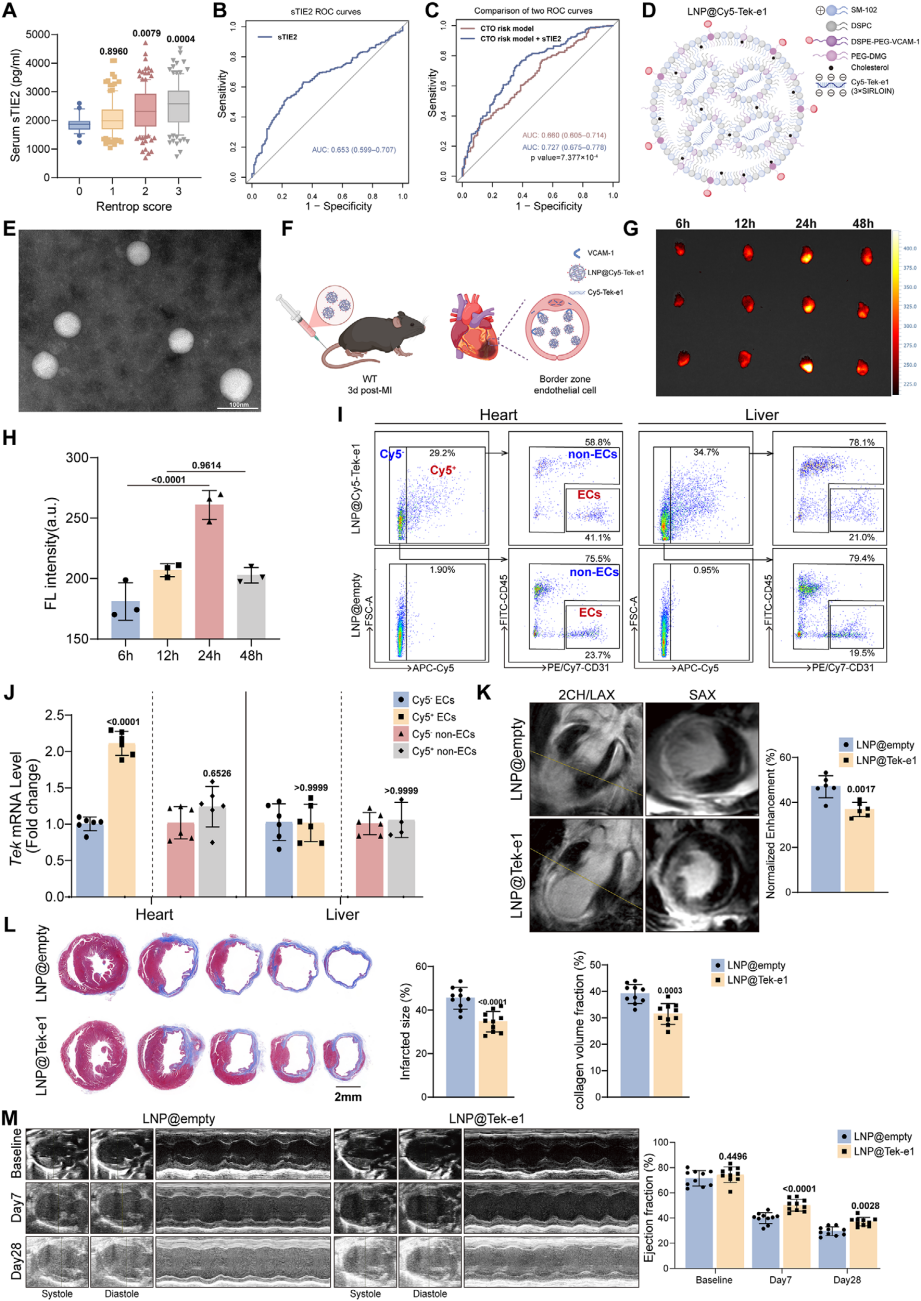
A clinically relevant TIE2 axis supports dual‐selective Tek‐e1 therapy for ischemic cardiac repair. (A) Serum soluble TIE2 (sTIE2) levels in patients with coronary chronic total occlusion stratified by Rentrop grade (Rentrop 0, n = 23; Rentrop 1, n = 127; Rentrop 2, n = 140; Rentrop 3, n = 112). (B) Receiver operating characteristic curve evaluating sTIE2 for discrimination between poor (Rentrop 0–1) and good (Rentrop 2–3) coronary collateral circulation. (C) Comparison of receiver operating characteristic curves for the clinical model alone and the clinical model supplemented with sTIE2. The clinical model included sex, age, body mass index, smoking, hypertension, diabetes, prior myocardial infarction, and serum creatinine. (D) Schematic of the SM‐102‐based, VCAM‐1‐targeted lipid nanoparticle encapsulating Cy5‐labeled Tek‐e1 carrying three mouse SIRLOIN nuclear‐enrichment elements. (E) Transmission electron microscopy analysis of LNP@Cy5–Tek‐e1. (F–H) Cardiac accumulation of LNP@Cy5–Tek‐e1 following intravenous administration on day 3 after MI. (F) Schematic of LNP administration and targeting of border‐zone endothelial cells. Created in BioRender. Yang, L. (2026) https://BioRender.com/4cwb5c8. (G) Ex vivo fluorescence imaging of hearts at 6, 12, 24, and 48 h after injection. (H) Quantification of cardiac fluorescence intensity (n = 3 mice per time point; one‐way ANOVA with Tukey's multiple‐comparisons test). (I, J) Cellular distribution and transcriptional activity of LNP@Cy5–Tek‐e1 after 1 week of treatment. (I) Flow‐cytometric analysis of Cy5^+^ and Cy5^−^ endothelial and non‐endothelial cell populations in the heart and liver following treatment with LNP@Cy5–Tek‐e1 or LNP@empty. (J) RT–qPCR analysis of *Tek* mRNA in sorted Cy5^−^ endothelial cells, Cy5^+^ endothelial cells, Cy5^−^ non‐endothelial cells, and Cy5^+^ non‐endothelial cells from the heart and liver (n = 6 per group; two‐way ANOVA with Sidak's multiple‐comparisons test). (K) Late gadolinium enhancement magnetic resonance imaging in two‐chamber long‐axis and short‐axis views and quantification of normalized enhancement in the infarct border zone following LNP@empty or LNP@Tek‐e1 treatment (n = 6 mice per group; unpaired two‐tailed Student's *t*‐test). (L) Masson's trichrome staining of serial short‐axis heart sections and quantification of infarct size and collagen volume fraction on day 28 after MI (n = 10 mice per group; unpaired two‐tailed Student's *t*‐test). (M) M‐mode echocardiography and quantification of left ventricular ejection fraction at baseline and on days 7 and 28 after MI (n = 10 mice per group; two‐way ANOVA with Sidak's multiple‐comparisons test). CTO, chronic total occlusion; sTIE2, soluble TIE2; AUC, area under the curve; LNP, lipid nanoparticle; MI, myocardial infarction; EC, endothelial cell; 2CH/LAX, two‐chamber long‐axis view; SAX, short‐axis view. Boxes in (A) indicate the median and interquartile range. Other quantitative data are presented as mean ± SD. Each dot represents an individual patient, mouse, or independent biological sample, as appropriate.

**TABLE 1:** Association of Rentrop score with sTIE2.

	sTIE2 < 2163.75pg/mL	sTIE2 ≥ 2163.75pg/mL	*p* value
Unadjusted	1 (Ref.)	2.990 (1.968–4.588)	< 0.001
Model 1	1 (Ref.)	2.999 (1.972–4.605)	< 0.001
Model 2	1 (Ref.)	3.325 (2.112–5.315)	< 0.001

Model 1: adjusted for age and sex.

Model 2: Model 1+diabetes+hypertension+body mass index + smoking + prior myocardial infarction + serum creatinine

The area under the receiver operating characteristic curve for sTIE2 discrimination of collateral status was 0.653 (95% CI 0.599–0.707, P < 0.001), with an optimal cutoff of 2346.00 pg/mL, yielding a sensitivity of 52.78% and a specificity of 76.67% (Figure 7B). Adding sTIE2 to a clinical model comprising sex, age, body mass index, smoking, hypertension, diabetes, prior myocardial infarction, and serum creatinine increased the area under the curve from 0.660 to 0.727 (P < 0.001; Figure 7C). Continuous net reclassification improvement was 0.653 (95% CI 0.463–0.842), and integrated discrimination improvement was 0.067 (95% CI 0.044–0.090; both P < 0.001; Table 2). Thus, sTIE2 was independently associated with well‐developed coronary collateral circulation and provided incremental discriminatory value beyond conventional clinical variables, supporting an association between the TIE2 axis and ischemic coronary adaptation in humans.

**TABLE 2:** Comparison of the Predictive Value of sTIE2 on CTO Risk Model for Rentrop score using AUC, NRI and IDI.

	AUC	*p* value	Continuous NRI (95% CI)	*p* value	IDI (95% CI)	*p* value
CTO risk model vs. model+sTIE2	0.660 vs. 0.727	< 0.001	0.653 (0.463 – 0.842)	< 0.001	0.067 (0.044 – 0.090)	< 0.001

AUC, area under the curve; NRI, net reclassification improvement; IDI, integrated discrimination improvement.

Genetic and pharmacological studies in mice established that TIE2 is required for microvascular homeostatic restoration, while the patient cohort further supported the clinical relevance of this pathway in human ischemic vascular adaptation. We therefore investigated whether Tek‐e1 could be used to enhance endogenous *Tek* transcription. Tek‐e1 acts on the endogenous *Tek* promoter–enhancer unit, and its activity depends on the functional state of the enhancer, thereby conferring intrinsic cell‐ and state‐dependent transcriptional responsiveness.

Accordingly, we generated SM‐102‐based lipid nanoparticles encapsulating Cy5‐labeled Tek‐e1, termed LNP@Cy5–Tek‐e1 (Figure 7D). The nanoparticle surface was functionalized with a DSPE–PEG‐conjugated VCAM‐1‐targeting ligand to promote uptake by activated endothelial cells after MI. Capped Tek‐e1 was fused to three mouse SIRLOIN nuclear‐enrichment elements to increase nuclear accumulation and facilitate its activity at the *Tek* locus. Microfluidic mixing produced uniform nanoparticles with a mean diameter of approximately 100 nm and an RNA loading capacity of approximately 4.5% (w/w) (Figure 7E). This system therefore integrated injury‐associated endothelial targeting, nuclear RNA enrichment, and *Tek* locus state‐dependent transcriptional responsiveness.

Following intravenous administration of LNP@Cy5–Tek‐e1 on day 3 after MI, Cy5 signals were detected predominantly in the heart and liver but displayed distinct temporal kinetics. Liver fluorescence peaked at 6–12 h and subsequently declined, whereas cardiac fluorescence peaked at 24 h and decreased by 48 h (Figures 7F–H and S9B,C). The early hepatic peak was consistent with rapid uptake of circulating LNPs, whereas the delayed cardiac peak was consistent with progressive accumulation in the injured myocardium. Based on these kinetics, LNP@TEK‐e1 was administered every 48 h for 1 week beginning on day 3 after MI.

The hepatic safety and systemic inflammatory response to LNP@Tek‐e1 were also evaluated. Mice received intravenous injections on days 3, 5, and 7 after MI, and blood samples were collected 4 h after the final injection on day 7 and on day 14. LNP@Tek‐e1 treatment did not significantly alter serum ALT or AST levels (Figure S9D), nor did it further elevate circulating IFN‐α or TNF‐α levels following MI (Figure S9E). Although IL‐6 levels increased modestly on day 7, they returned to levels comparable to those of PBS‐treated controls by day 14 (Figure S9E). These findings support a favorable biosafety profile for LNP@Tek‐e1.

After 1 week of treatment, Cy5^+^ cells were detected predominantly in the heart and liver. Flow cytometry showed that, in the heart, CD45^−^CD31^+^ endothelial cells were enriched within the Cy5^+^ population relative to the Cy5^−^ population, whereas no comparable endothelial enrichment was observed in the liver (Figures 7I and S9F,G). Further sorting according to Cy5 status and cell identity showed that *Tek* mRNA was significantly increased only in Cy5^+^ cardiac endothelial cells, with no detectable change in Cy5^−^ cardiac endothelial cells, cardiac non‐endothelial cells, hepatic endothelial cells, or hepatic non‐endothelial cells (Figure 7J).

These findings distinguish nanoparticle exposure from the functional transcriptional response to Tek‐e1. LNP uptake alone was insufficient to induce *Tek* expression; instead, the transcriptional response was largely restricted to cardiac endothelial cells that had internalized Tek‐e1. Thus, VCAM‐1‐mediated targeting provided delivery‐level selectivity, whereas the dependence of Tek‐e1 on a permissive *Tek* locus further constrained its transcriptional output, together establishing a dual‐selection mechanism.

Finally, we evaluated the therapeutic effects of LNP@Tek‐e1. Compared with LNP@empty, LNP@Tek‐e1 significantly increased the proportions of NG2^+^ pericyte‐covered microvessels and VE‐cadherin^+^ microvessels in the infarct border zone (Figure S10A, B), accompanied by reduced FITC–dextran leakage and increased microvascular luminal diameter (Figure S10C, D). Consistent with these findings, normalized late gadolinium enhancement in the infarct border zone was significantly reduced (Figure 7K), supporting enhanced microvascular maturation and stability, alongside improved local microvascular function. By day 28 after MI, LNP@Tek‐e1 reduced infarct scar size and collagen volume fraction (Figure 7L). Echocardiography further showed higher ejection fractions on days 7 and 28, together with attenuated late left ventricular dilation, in the LNP@Tek‐e1 group (Figures 7M and S10E).

Collectively, the CTO cohort identified higher circulating sTIE2 as an independent marker of well‐developed coronary collateral circulation and demonstrated its incremental value for discriminating collateral status, supporting a role for the TIE2 axis in human ischemic vascular adaptation. Leveraging the dependence of Tek‐e1 on a permissive endogenous *Tek* locus, we developed a delivery strategy integrating injury‐associated endothelial targeting, nuclear enrichment, and locus‐state gating. LNP@Tek‐e1 selectively increased *Tek* expression in cardiac endothelial cells that internalized Tek‐e1 and improved microvascular perfusion, ventricular remodeling, and cardiac function after MI. This combination of delivery selectivity and endogenous cis‐regulatory context dependence provides a mechanism‐matched therapeutic strategy for reinforcing the Tek‐e1–TIE2 reparative program.

## 3 Discussion

This study identifies hypoxia‐induced endothelial KDM5A downregulation as an endogenous epigenetic response that favours microvascular homeostatic repair rather than angiogenic initiation after MI. KDM5A loss establishes focal H3K4me3 enrichment at the *Tek* locus, permitting ISL1‐dependent coactivation of *Tek* and Tek‐e1, which reinforces enhancer–promoter communication through R‐loop formation and is required for full *Tek*/TIE2 induction. This KDM5A–ISL1–Tek‐e1–TIE2 axis promotes microvascular maturation, barrier integrity and border‐zone perfusion, thereby limiting adverse left ventricular remodelling.

In *Kdm5a* ^cKO^ mice, at day 7 after MI, we observed improved cardiac function, increased border zone perfusion, and attenuated adverse left ventricular remodeling. These findings indicate that endothelial *Kdm5a* deletion enhances revascularization in the border zone. Unexpectedly, conventional indices of angiogenesis did not differ significantly, whereas local microvascular immunofluorescence showed enlarged luminal caliber. Further analyses revealed features of microvascular homeostasis in *Kdm5a* ^cKO^ mice, including increased pericyte coverage, larger lumens, reduced leakage, and decreased endothelial cell apoptosis. A large body of evidence indicates that lumen size and vascular maturation, rather than capillary number per se, are key determinants of effective perfusion and prognosis [34, 35, 36, 37]. Our data suggest that restoration of microvascular homeostasis via endothelial KDM5A targeting is a more effective approach to revascularization.

KDM5 enzymes are 2‐oxoglutarate/Fe(II)–dependent demethylases that specifically remove H3K4me3/2 marks and are highly sensitive to oxygen and metabolites [38]. Hypoxia can directly inhibit their catalytic activity or reduce their expression [13, 14]. Western blot analysis of endothelial cells isolated from the infarct border zone revealed changes only in the abundance of H3K4me3/2, with a predominant increase in H3K4me3. This narrow and locus‐specific pattern is more consistent with the specific, reversible turnover of H3K4me3 mediated by the KDM5 family rather than writer upregulation leading to broad promotion of H3K4‐site methylation [39, 40].

Our findings further indicate that KDM5A regulation is highly locus‐specific and of substantial effect size. In endothelial cells, *Kdm5a* deletion leaves the global H3K4me3 landscape and peak width largely unchanged, while producing pronounced enrichment at only a small subset of genes, a “few‐but‐strong” local amplification pattern. Consistent with this, Sonia Rocha and colleagues reported that hypoxic inhibition of KDM5A activity elevates H3K4me3 at only a subset of genes, and that KDM5A loss reproduces this effect chiefly at those loci [13]. This pattern is compatible with a threshold‐gating role for KDM5A at key promoters or enhancers. Prior work also demonstrates that, in addition to its demethylase activity, the KDM5 family can influence transcription through noncatalytic mechanisms, including protein interactions and complex assembly [37, 40, 41, 42]. Using the KDM5 demethylase inhibitor KDM5A‐IN‐1, we found evidence for a catalysis‐dependent mechanism at the *Tek* locus [43]. Taken together, these studies, alongside our pharmacologic and genetic data, indicate that KDM5A primarily shapes H3K4me3 at critical loci through its catalytic activity, thereby enabling precise control of transcriptional output.

Upon endothelial *Kdm5a* deletion, the *Tek* promoter and its enhancer concurrently acquire marked enrichment of H3K4me3, thereby establishing a more permissive chromatin platform that facilitates the parallel assembly and stable engagement of the transcriptional machinery. Prior studies have shown that transcription at promoters and enhancers can be governed by the same transcription factors, which helps organize chromatin architecture [44, 45, 46]. Notably, another study also demonstrated that ISL1 binds both promoters and enhancers, thereby regulating the expression programs of lineage‐specific genes [47]. In our system, ISL1 occupancy was detected at both the *Tek* promoter and enhancer and was further strengthened in the H3K4me3‐enriched state induced by KDM5A loss. However, transcription‐factor co‐occupancy alone did not account for full *Tek*/TIE2 activation. eRNAs are more than by‐products of active enhancers and act as extensions of enhancer activity that boost transcription through multiple routes, most commonly by reinforcing enhancer–promoter looping [19]. Tek‐e1 acts as a necessary RNA component of this regulatory unit. Its GC‐rich, positively GC‐skewed sequence confers an intrinsic propensity for R‐loop formation, and Tek‐e1 rescue and RNase H1 experiments show that Tek‐e1‐dependent DNA:RNA hybrid formation is required to reinforce enhancer‐promoter communication and sustain full ISL1‐dependent *Tek*/TIE2 induction. Thus, the *Tek* locus is controlled by a two‐step epigenetic‐eRNA mechanism: KDM5A loss first establishes a permissive H3K4me3‐marked chromatin state that enables ISL1‐dependent coactivation of *Tek* and Tek‐e1, whereas Tek‐e1 subsequently stabilizes enhancer‐promoter communication through R‐loop formation to convert chromatin priming into full TIE2 transcriptional output.

Active enhancers and their eRNAs exhibit pronounced tissue‐ and cell‐type specificity [48]. Consistent with this, our nuclear RNA profiling across multiple organs and skeletal muscle detected Tek‐e1 only in the heart and lung. The pro‐transcriptional effects of eRNAs are highly dependent on the activation state of their source enhancers [19, 49], and this intrinsic specificity confers a substantial targeting advantage. Lipid nanoparticles (LNPs) have achieved clinical validation as delivery vehicles for both siRNA and mRNA modalities, establishing RNA therapeutics as a novel intervention across multiple diseases [50, 51]. Leveraging VCAM‐1 upregulation on inflamed endothelium, we engineered an LNP delivery system surface‐functionalized with a VCAM‐1–binding ligand [51]. This platform implements dual targeting—receptor‐directed enrichment and enhancer‐activity dependence—achieving greater precision and stronger biological effects in target cells. This approach provides a practical route to organ‐, cell‐, and locus‐level precision delivery and gene‐regulatory therapy in ischemic disease settings.

Our study demonstrates that TIE2 plays a critical role in revascularization after MI. Accumulating clinical evidence also suggests that sTIE2 may serve as a clinically relevant biomarker. However, the relationship between sTIE2 levels and tissue perfusion remains controversial across different disease settings. For example, a previous small‐scale study in peripheral artery disease reported that serum sTIE2 levels were markedly elevated in patients with severe limb ischemia compared with those with intermittent claudication or healthy controls [52]. In contrast, an earlier study in patients with heterogeneous coronary artery disease (CAD) reported that well‐developed CCV were associated with higher local sTIE2 levels in the coronary circulation [53]. Extending these observations, we investigated serum sTIE2 in a moderately sized, contemporary, and clinically more homogeneous cohort of patients with CTO. We further demonstrate that serum sTIE2 is independently associated with CCV formation, provides incremental predictive value beyond conventional risk models, and yields a quantitative cutoff for risk stratification, thereby establishing its clinical utility as a biomarker in this setting. Collectively, these findings identify serum sTIE2 as a novel biomarker for collateral assessment in CTO and provide the first evidence of its clinical relevance in this specific population.

Membrane‐bound TIE2 and circulating sTIE2 have distinct functional properties. sTIE2 is generated through proteolytic shedding of the TIE2 extracellular domain, a process regulated by PI3K–AKT signaling, suggesting that circulating sTIE2 may reflect endothelial TIE2 expression and receptor activation during vascular remodeling [54]. Although sTIE2 can inhibit TIE2 activation by sequestering ANGPT1, in vitro studies showed that approximately 40‐fold and 194‐fold molar excesses of sTIE2 over ANGPT1 were required to achieve 10% and 50% inhibition, respectively, substantially exceeding the ratios typically observed in circulation under physiological and pathological conditions [55]. Moreover, our findings demonstrate that KDM5A downregulation increases endothelial TIE2 expression, indicating that elevated circulating sTIE2 does not necessarily imply reduced membrane‐bound TIE2 abundance or signaling activity. Consistent with our clinical findings, Ju et al. reported a positive association between serum sTIE2 levels and cerebral collateral circulation assessed by digital subtraction angiography in patients with ischemic stroke, and further demonstrated that TIE2–PI3K–AKT signaling promotes collateral vascular remodeling and improves perfusion following ischemia [56]. These observations support circulating sTIE2 as a potential biomarker reflecting TIE2‐associated vascular remodeling during ischemia.

Dissecting the RNA‐specific function of Tek‐e1 remains challenging because of its genomic context. Tek‐e1 is transcribed in the same orientation as Tek and overlaps Tek pre‐mRNA; therefore, RNase H‐dependent Gapmer/ASOs may interfere with both Tek‐e1 abundance and host‐gene transcription [57]. Although our 20‐bp ΔTSS deletion lies outside the annotated enhancer interval, potential effects on local cis‐regulatory activity cannot be excluded. RNA add‐back and nuclear‐targeted RNase H1 experiments provide complementary evidence supporting the involvement of Tek‐e1 RNA and R‐loop formation in enhancer–promoter communication. Nevertheless, more selective perturbation strategies are needed to fully distinguish the contributions of the RNA molecule, the act of transcription, and local DNA regulatory elements.

## 4 Conclusion

This study identifies a hypoxia‐responsive hypertranscriptional endothelial state in the infarct border zone that preferentially supports restoration of microvascular homeostasis. KDM5A downregulation primes the *Tek* locus for ISL1‐dependent coactivation of *Tek* and Tek‐e1, with Tek‐e1 required to reinforce enhancer–promoter communication through R‐loop formation and achieve full *Tek*/TIE2 induction. These findings identify Tek‐e1‐mediated transcriptional reinforcement as a strategy for amplifying endogenous vascular repair after cardiac ischaemic injury.

## 5 Experimental Section/Methods

A detailed description of our methods is provided in the Supplemental Material.

### 5.1 Sex as a Biological Variable

Our study examined male mice because male animals exhibited less variability in phenotype. It is not known whether these findings are relevant for female mice. For human studies, data were collected from both men and women.

### 5.2 Animals

Endothelial‐specific *Kdm5a* deletion was achieved by crossing *Cdh5*‐Cre; *Kdm5a* ^fl/fl^ mice, and littermate *Cdh5* ^Cre^ mice served as controls. All mice used in this study (including routine C57BL/6, *Cdh5* ^Cre^ and *Cdh5*‐Cre; *Kdm5a* ^fl/fl^ strains) were purchased from Cyagen (Cyagen Biosciences Inc., Suzhou, China). Mice were housed in a specific pathogen–free facility under a 12‐hour light/dark cycle, with controlled temperature and humidity, and were provided ad libitum access to food and water. All experimental procedures were approved by the Animal Care Ethics Committees of Second Affiliated Hospital, Zhejiang University (2024‐008; 2025‐089) and were conducted in accordance with the National Institutes of Health Guide for the Care and Use of Laboratory Animals. In accordance with the ARRIVE guidelines, animals were randomly allocated to experimental groups and outcomes were assessed in a blinded manner.

### 5.3 Statistical Analysis

Experimental data (cell and animal) were expressed as the mean ± standard deviation (SD) of at least three biological replicates. Data normality was estimated using the Shapiro‐Wilk test. According to the normality, the Student's t‐test was performed to determine differences between two groups, and the One‐Way ANOVA test was performed to determine the differences among three or more groups. When studying the effects of two variables, Two‐way ANOVA was appropriate. Tukey or Sidak post hoc multiple comparison test was used for both One‐ and Two‐Way ANOVA tests. Two‐sided P < 0.05 indicated significance. The statistical analyses were performed using SPSS 18.0 and GraphPad Prism 9.

Statistical analysis of clinical data used R version 4.0.2 and SPSS 18.0. Normally distributed continuous variables were expressed as mean ± SD; non‐normally distributed variables as median and interquartile range. Categorical data were shown as absolute values and percentages, analyzed using chi‐square or Fisher's exact test. Group differences were determined using Student's t‐test or Wilcoxon rank‐sum test. Kruskal‐Wallis test with Dunn's multiple comparison was used to assess serum sTIE2 levels across Rentrop scores 0, 1, 2, and 3. Logistic regression analysis evaluated the association between sTIE2 and CCV. Model 1 adjusted for age and sex; Model 2 added BMI, smoking, hypertension, diabetes, previous myocardial infarction, and serum creatinine. ROC curve analysis assessed the predictive value of sTIE2 for CCV. Area under the curve (AUC), continuous net reclassification improvement (NRI), and integrated discrimination improvement (IDI) compared sTIE2's discriminative ability in traditional CTO risk models. DeLong's method compared AUC differences. Spearman correlation analysis explored the relationship between sTIE2 and CCV. All tests were two‐tailed, with p < 0.05 considered statistically significant.

### 5.4 Study Approval

All mouse studies were approved by the Animal Care and Use Committee of Second Affiliated Hospital, Zhejiang University School of Medicine (2024‐008; 2025‐089). All participants provided written informed consent, and was approved by the Fujian Provincial Hospital Ethics Committee, and the protocol was carried out in accordance with the Declaration of Helsinki (K2017‐12‐025).

## Author Contributions

**Yang Lyu**: conceptualization, methodology, software, data curation, validation, investigation, writing – original draft, visualization. **Kun Yang**: conceptualization, investigation, writing – review and editing, funding acquisition, project administration, resources, supervision. **Yunzhi Ling**: validation, visualization, writing – original draft. **Jiahui Cheng**: validation, software, data curation. **Jiaran Shi**: funding acquisition, project administration, resources. **Shiyu Hu**: investigation, validation, software, data curation. **Changyi Zhou**: methodology. **Wei Wei**: investigation, validation. **Hanchuan Chen**: software, data curation. **Xiang Wei**: investigation, validation. **Zengru Wang**: investigation, validation, formal analysis. **Zhi Xiong**: formal analysis, data curation. **Chenxi Yang**: investigation. **Su Zou**: investigation. **Rui Shen**: investigation, funding acquisition. **Yuanyue Song**: investigation. **Gaofeng Zhang**: resources, project administration, writing – review and editing, supervision. **Rifeng Gao**: supervision, resources, project administration, writing – review and editing. **Kaiyang Lin**: supervision, resources, project administration, funding acquisition, writing – review and editing. **Yingjia Xu**: supervision, resources, funding acquisition, writing – review and editing.

## Funding

Leading Talent Program of Minhang District of Shanghai (Grant number:202205, to Y.X.); The Youth Top Talent Project of Fujian Provincial Foal Eagle Program (no grant number, to K.L.); Fujian Research and Training Grants for Young and Middle‐aged Leaders in Healthcare (no grant number, to K.L.); The Natural Science Foundation of Shanghai (Grant number: 24ZR1448200, to K.Y.); The Youth Program of the National Natural Science Foundation of China (Grant number: 82400395, to K.Y.); The Youth Program of the National Natural Science Foundation of China (Grant number: 82500319, to R.G.); The Natural Science Foundation of Zhejiang Province (Grant number: LQN26H020005, to J.S.); The Natural Science Foundation of Ningbo (Grant number: 2025J055, to J.S.); The Health Science and Technology Program of Zhejiang Province (Grant number: 2025HY0894, to J.S.); Ningbo Key Research and Development Program (Grant number: 2024Z232, to J.S); The 2025 Key Specialty Development Program of Shanghai Fifth People's Hospital (Grant number: 2025WYZDZK06, to G.Z.).

## Conflicts of Interest

The authors declare no conflicts of interest.

## Supporting information




**Supporting File**: advs78650‐sup‐0001‐SuppMat.docx.

## Data Availability

The data that support the findings of this study are available from the corresponding author upon reasonable request.
