# Supplementary material for: Cardiac Endothelial Enhancer RNA Tek‐e1 Restores Microvascular Homeostasis After Myocardial Infarction
Source: Adv Sci (Weinh). 2026 Nov 9:e78650. Online ahead of print. doi: 10.1002/advs.78650 (PMC13748899; doi:10.1002/advs.78650)
Supplement: Supplementary file 1 [file ADVS-9999-e78650-s001.docx]

Supporting Information

**Cardiac endothelial enhancer RNA Tek-e1 restores microvascular homeostasis after myocardial infarction**

*Yang Lyu^1,2†^, Kun Yang^3*†^, Yunzhi Ling^4†^, Jiahui Cheng^6†^, Jiaran Shi^7^, Shiyu Hu^8^, Changyi Zhou^8^, Wei Wei^9^, Hanchuan Chen^10^, Xiang Wei^11^, Zengru Wang^1,2^, Zhi Xiong^12^, Chenxi Yang^13^, Su Zou^2^, Rui Shen^14^, Yuanyue Song^2^, Gaofeng Zhang^1^, Rifeng Gao^5*^, Kaiyang Lin^4*^, Yingjia Xu^2 *^*

**Supplementary Methods**

**Mouse model of myocardial ischemia**

Mice were subjected to myocardial infarction (MI) or sham surgery under isoflurane anesthesia. For MI, mice were anesthetized with 1–2% isoflurane, intubated, and mechanically ventilated. Following a left thoracotomy at the third intercostal space, the left anterior descending coronary artery (LAD) was permanently ligated with a 7-0 silk suture. Visible blanching of the myocardium distal to the ligation was used as an indicator of successful occlusion. Sham-operated mice underwent the same procedure except that the LAD was not ligated. Perioperative analgesia and body temperature maintenance were provided according to institutional guidelines.

**Application of KDM5A-IN-1, Compound 5, COMP-Ang1, and LNP@Cy5–Tek-e1**

KDM5A-IN-1, Compound 5, and LNP@Cy5–Tek-e1 were administered to mice as indicated. For KDM5A-IN-1, mice received KDM5A-IN-1 (5 mg/kg, i.p.) once daily starting on the day of MI surgery. For Compound 5, mice received Compound 5 (25 mg/kg, i.p.) every 2 days starting on the day of MI surgery. For COMP-Ang1, mice received COMP-Ang1 (30 μg/mouse, i.p.) every 2 days starting on the day of MI surgery. For LNP, mice received 0.5mg/kg RNA dose per injection via tail-vein (i.v.) every 2 days for 1 week, beginning on day 3 post-MI. Vehicle-treated and LNP@empty–treated mice served as controls.

**Echocardiography analysis**

Cardiac function was evaluated by transthoracic echocardiography using a Vevo 3100 high-frequency ultrasound system (VisualSonics). Mice were anesthetized with isoflurane (3% induction; 1.5% maintenance), and positioned on a warmed platform with ECG monitoring. Heart rate was maintained at 400–550 beats per minute throughout imaging. M-mode images were collected for quantitative assessment. Measurements were averaged over 3–5 consecutive cardiac cycles and analyzed using Vevo LAB software by an investigator blinded to group allocation.

**MRI measurements.**

All MRI experiments were performed using a horizontal bore 9.4T/30 cm uMR (United Imaging Life Science Instrument, Wuhan, China). An 86-mm diameter transmit volume coil and a three-loop surface coil were used. A combination of an 86-mm inner diameter (ID) volume coil for radio frequency (RF) transmission and a custom-designed dedicated RF coil for mice cardiac imaging. This coil was positioned on the mouse's abdomen to encompass the thoracic region containing the heart. To synchronize data acquisition with the ECG signs, ECG triggering gating (SA Instruments) were applied. Typical heart rates were between 375– 500 bpm (cycle length 120 to 160 msec) during imaging^1^. Mice were initially induced to anesthesia by 4% isoflurane. Then mice were subsequently transferred to the MRI bed with heating systems and fixed by bite and ear bars. Isoflurane level was then gradually reduced and maintained at 1.2%. During this experiment, animals were maintained at a light and stable level of anesthesia and the rectal temperature was monitored and kept at 37.0 °C (SA Instruments)^2^. A cardiac gated GRE sequence was used to acquire cardiac CINE images. Imaging parameters for mice were as follows: TR/TE=2.90/1.12 ms, FOV =16×32 mm^2^, matrix size =104×208, with 6 slices which thickness was 1.0 mm. A single short-axis slice was obtained in approximately 1.5 mins, leading to a total scan time for cine data of around 10 minutes. For delayed contrast enhancement 0.6 mmol/kg Gd-DOTA was injected, via an intra-peritoneal route for mice, following initial baseline scanning. Following contrast agent administration and short time delay of ~20 minutes to allow for late enhancement to establish a high SNR multi-slice IR GRE sequence with single TI point was used to acquire late gadolinium enhancement (LGE) images. Additional parameters for mice: TR/TE=3.01/1.17 ms, FOV=16×32 mm^2^, matrix size=136×272, slice thickness = 1.0 mm, flip angle=30°, number of signal average=4, TI=250 ms, total acquisition time ~3.5 minutes/slice^3^.

Late gadolinium enhancement (LGE) images were analyzed using ImageJ (NIH) by an investigator blinded to group allocation. Regions of interest (ROIs) were manually drawn on short-axis slice to measure mean signal intensity (SI) in the border zone myocardium and in the remote myocardium within the same slice, avoiding the ventricular blood pool and obvious artifacts. Normalized enhancement (NE, %) was calculated as: NE (%) = (SI_border − SI_remote) / SI_remote × 100.

**Triphenyltetrazolium chloride (TTC) staining**

After 12 h of MI, mice were anesthetized with 1% pentobarbital (70 mg/kg). Then, the heart was quickly excised and rinsed by placebo saline solution and immediately frozen at -80 °C for 30 min. The frozen heart was quickly sliced into 5 1-mm–thick short-axis sections and incubated in 1% TTC solution at 37 °C for 30 min. Each section was flattened and fixed with 4% paraformaldehyde. TTC-negative (unstained/pale) myocardium was defined as the infarcted area.

**Masson’s trichrome staining**

Masson’s trichrome staining was used to assess infarct size and collagen deposition in hearts. Tissues were fixed in 4% paraformaldehyde for 24 h at room temperature, paraffin-embedded, and sectioned at 3 μm. Stained with Masson’s trichrome according to the manufacturer’s instructions. Infarct size and collagen volume fraction were quantified by light microscopy using ImageJ. Infarct size was expressed as a percentage of the left ventricular (LV) circumference, and collagen volume fraction was calculated as the trichrome-positive area normalized to total myocardial area.

**Multiplex immunohistochemical (mlHC) staining using Tyramide Signal Amplification (TSA)**

Multiplex immunohistochemistry was performed using a TSA-based Bas Multi-target Immunohistochemistry Kit (AXB, BXT35010011). Paraffin sections were deparaffinized, rehydrated through graded alcohol, rinsed in distilled water, and subjected to antigen retrieval in EDTA buffer. Slides were blocked with antibody diluent/blocking reagent and incubated with primary antibodies for 1 h, followed by HRP-labeled goat anti-(mouse/rabbit) reagent for 10 min at 37°C. Fluorophore tyramide substrates were applied for visualization. After each staining cycle, heat-induced epitope retrieval was used to strip prior antibodies. Slides were counterstained with DAPI for 5 min and mounted in antifade medium. Primary antibodies included anti-CD31 (Abcam, ab9498), anti-CD45 (Abcam, ab318154), anti-KDM5A (Abcam, ab194286), anti-cTnT (Abcam, ab209813), anti-VE-cadherin (Abcam, ab33168), anti-NG2 (Abcam, ab275024). Fluorescence was quantified in 10 random fields per section.

**In vivo permeability assessment**

Cardiac vascular permeability was quantified using FITC–dextran leakage. Mice were injected via the tail vein with 100 μL FITC–dextran (70 kDa; 50 mg/mL; Sigma, 46945) 30 min prior to euthanasia. Hearts were perfused with ice-cold PBS to remove intravascular tracer, embedded in OCT compound, rapidly frozen, and sectioned on a cryostat (8μm thickness). Nuclei were counterstained with DAPI. Fluorescent images were captured using a Leica microscope, and FITC signal intensity was quantified in 10 randomly selected high-power fields (40×) per section using ImageJ.

**Microsphere-based assessment of microvascular lumen patency**

Mice were anesthetized and cannulated via the left ventricle. A needle was inserted into the left ventricle, and the circulation was perfused with 5 mL warm heparinized saline using a peristaltic pump (2 mL/min). The aortic arch was clamped to facilitate delivery of microspheres into the coronary circulation, and 250 μL of microsphere suspension was infused (0.5 mL/min). The suspension contained 5 × 10⁴ 4-μm FITC–carboxylate microspheres, 2 mM EDTA, 0.01% Tween-20, 10 U/mL heparin, and 0.1% BSA. Subsequently, 1 mL warm heparinized saline was infused (1 mL/min) to flush residual microspheres. The left ventricular anterior wall was collected, embedded in OCT compound, rapidly frozen, and longitudinally cryosectioned at 8 μm thickness. Sections were immunostained for CD31, followed by Alexa Fluor–conjugated secondary antibodies. Images were acquired using a Leica fluorescence microscope. Quantification was performed in the border zone using 10 randomly selected fields per section (20×) in ImageJ. Bead-occluded vessels (%) were calculated as the number of CD31⁺ microvessels containing ≥1 entrapped microsphere divided by the total number of CD31⁺ microvessels.

**Flow cytometry**

Single-cell suspensions were prepared from the heart, liver, lung, kidney, and brain after intracardiac perfusion with ice-cold PBS. Tissues were minced and enzymatically digested, followed by filtration through a 70-μm cell strainer and washing in FACS buffer. Dead cells were excluded using Zombie Aqua™ Fixable Viability Dye (BioLegend, 423102). Fc receptors were blocked with TruStain FcX™ (BioLegend, 101320) for 15 min at 4°C, followed by surface antibody staining for 30 min at 4°C. For intracellular/nuclear staining, cells were fixed using the FOXP3 Fix/Perm Buffer Set (BioLegend, 4421403) for 20 min at room temperature in the dark, permeabilized with Perm/Wash buffer for 15 min at room temperature, and then incubated with intracellular/nuclear antibodies for 30 min at room temperature in the dark. Flow cytometry panels were configured to identify CD45⁻CD31⁺ endothelial cells, using anti-CD45 (BioLegend, 103154), anti-CD31 (BioLegend, 102524), and anti-Ki-67 (BioLegend, 652411). Data were acquired on a BD FACSAria™ III and analyzed using FlowJo v10.

**Isolation and culture of mouse cardiac endothelial cells and pericytes**

Cardiac microvascular endothelial cells (pCMECs) and pericytes (PCs) were isolated from hearts of 8-week-old mice. Hearts were minced and digested for 30 min in an enzyme mixture containing collagenase II (1 mg/mL), collagenase IV (2.5 mg/mL), Dispase (0.25 U/mL), and DNase I (7.5 μg/mL). The cell suspension was filtered through a 70-μm cell strainer and washed. Endothelial cells were enriched by negative selection using CD45 MicroBeads (Miltenyi Biotec, 130-052-301) followed by positive selection using CD31 MicroBeads (Miltenyi Biotec, 130-097-418) according to the manufacturer’s instructions. For pericyte isolation, the CD45^-^CD31^-^ fraction was collected and stained with anti-NG2-PE antibody (abcam, ab317593), followed by positive magnetic selection using Anti-PE MicroBeads (Miltenyi Biotec, 130-048-801). pCMECs were cultured in 6-cm dishes in endothelial cell medium (ScienCell, 1001) supplemented with 5% fetal bovine serum, 100 U/mL penicillin, 100 μg/mL streptomycin, and 1% ECGF at 37°C and 5% CO2. Isolated pericytes were cultured in Pericyte Medium (ScienCell, 1231) under the same temperature and CO₂ conditions. Cells were used at early passages.

**Cell intervention**

For hypoxia experiments, cells were incubated in medium containing 1% FBS in a tri-gas incubator (Thermo Scientific, 3131) at 37°C, 5% CO₂, and 1% O₂ (balanced with N₂) for 24 h. Recombinant angiopoietin-1 (rANGPT1; 200 ng/mL) was then added for 30 min to assess acute TIE2 pathway phosphorylation, or for 12 h to evaluate sustained effects on endothelial junctional signaling/protein expression.

**Endothelial cell–pericyte coculture**

A non-contact Transwell coculture system was established to investigate the effects of primary cardiac pericytes on endothelial barrier function, apoptosis, and network stability. For permeability assays, endothelial cells were seeded onto Transwell inserts, while pericytes were cultured in the lower chambers. FITC–dextran permeability was assessed after 36 h of coculture. For apoptosis assays, endothelial cells were cultured in the lower chambers with pericytes in the upper inserts, followed by 12 h of hypoxic exposure and Annexin V-FITC/PI staining. For network stability assays, endothelial cells were seeded onto Matrigel-coated plates and allowed to form tubular networks for 6 h, after which pericyte-containing Transwell inserts were introduced. Network regression was assessed after an additional 12 h of coculture by quantifying the number of intact loops per field.

**Spheroid-based sprouting angiogenesis assay**

Endothelial sprouting was assessed using a spheroid-based assay. pCMECs (8 × 10^4^) were resuspended in 4 mL endothelial growth medium and mixed with 1 mL methylcellulose. The suspension was dispensed as 25-μL hanging drops onto the lid of a 10-cm dish and incubated inverted for 24 h to form spheroids. Spheroids were collected, washed with PBS, and embedded in collagen I gel. Collagen type I (Corning, 354236) was mixed on ice with 10× Medium 199 (Sigma, M9163), and pH was adjusted to 7.4 using 0.1 M NaOH (final collagen concentration, 2 mg/mL). The spheroid–collagen mixture was plated into 3.5-cm glass-bottom dishes (Cellvis, D35-20-1-N) and polymerized at 37°C for 30 min. VEGF (25 ng/mL) was added after gelation, and spheroids were incubated for 18 h before fixation in 4% paraformaldehyde and staining with Phalloidin (Solarbio, CA1610). Images were collected using a confocal microscope (Olympus FV3000RS). Sprout number and sprout length (from spheroid edge to tip) were quantified in 10 random fields per independent experiment using ImageJ.

**Tube formation and network regression assay**

Matrigel (Corning, 356234) was solidified in 24-well plates for 30 min at 37°C. pCMECs (1×10^5^ per well) were seeded in 1 mL endothelial cell medium and incubated for 6 h, followed by calcein staining (Solarbio, C7600) prior to imaging. Tube formation was quantified by the number of branch points and total tube length. To assess network stability, the established networks were further cultured for 12 h, with images acquired at 0 and 12 h after initial network formation. The number of intact loops per field was quantified using ImageJ.

**Transendothelial permeability assay**

Endothelial cells were seeded onto Transwell inserts and cultured for 36 h to establish an endothelial monolayer. Subsequently, 70-kDa FITC–dextran (Sigma, 46945) was added to the upper chamber at a final concentration of 250 μg/mL. After incubation for 2 h at 37°C, medium from the lower chamber was collected, and fluorescence intensity was measured using a fluorescence microplate reader. Endothelial permeability was expressed as relative FITC–dextran fluorescence intensity.

**Endothelial apoptosis assay**

Endothelial cells were cultured under hypoxic conditions (1% O₂) for 12 h. Cells were then collected and stained using an Annexin V-FITC/propidium iodide (PI) apoptosis detection kit (Beyotime, C1062M) according to the manufacturer's instructions. Apoptotic cells were quantified by flow cytometry as the percentage of Annexin V-positive cells.

**RNA transfection**

Primary cardiac microvascular endothelial cells (pCMECs) were seeded on gelatin-coated plates and transfected at ~60–70% confluence using CALNP® RNAi Transfection Reagent (DN001-10, DonaGene) according to the manufacturer’s instructions. Briefly, siRNA (final 75 nM) and CALNP® RNAi reagent were separately diluted in serum-free medium, combined, and incubated for 10–15 min at room temperature to allow complex formation. Complexes were then added dropwise to cells maintained in antibiotic-free complete medium. Medium was replaced 4–6 h later (or the next morning), and cells were harvested 24–48 h post-transfection for RT–qPCR and/or immunoblotting. A non-targeting siRNA was used as the negative control.

pCMECs were transfected with Tek-e1 RNA using CALNP™ mRNA in vitro Transfection Reagent (DN002-10, DonaGene) according to the manufacturer’s instructions. Reagent A and Reagent B were equilibrated to room temperature and vortexed. Transfection complexes were prepared in RNase-free tubes by strictly adding Tek-e1 RNA (final concentration of 1 µg/mL) first, followed by Reagent A, then Reagent B, with gentle mixing after each addition. After a 10-min incubation at room temperature, culture medium was added to reach the desired volume, and the complexes were added to cells. Medium was not changed within the first 24 h; for extended culture, fresh medium was replaced at 24 h post-transfection.

**In vitro transcription of Tek-e1**

The target sequence was cloned into the GBG IVT vector, linearized by BsaI digestion, and gel-purified. Using the linearized DNA (~25 μg) as the template, a 1-mL T7 in vitro transcription (IVT) reaction was assembled containing 1× transcription buffer; an NTP mixture (5 mM each, including Cy5-UTP); CleanCap (4 mM; co-transcriptional capping); RNase inhibitor (1 U/μL); yeast inorganic pyrophosphatase (0.002 U/μL); and T7 RNA polymerase (final 8 U/μL). The reaction was incubated at 37°C for 1 h, followed by DNase I treatment to remove the DNA template. RNA was purified according to the manufacturer’s instructions and validated under denaturing conditions to confirm transcript length and integrity. The resulting product was a 5′-capped, poly(A)-free, Cy5-labeled RNA, which was quantified in RNase-free water, stored at −80°C, and protected from repeated freeze–thaw cycles.

**Plasmid transfection**

Primary endothelial cells were transfected with plasmid DNA using Lipofectamine LTX Reagent with PLUS Reagent (Thermo Fisher Scientific) following the manufacturer’s protocol optimized for primary HUVECs. Cells were seeded one day prior to transfection to reach ~50–80% confluency. For each well of a 24-well plate, 0.5 μg endotoxin-free plasmid DNA was diluted in 100 μL Opti-MEM. PLUS Reagent (0.5 μL) was added to the diluted DNA and incubated for 5–15 min at room temperature. Lipofectamine LTX (typically 1.25–1.5 μL; optimization range 0.75–2.75 μL) was then added, followed by incubation for 25 min to allow complex formation. Cells were maintained in antibiotic-free complete growth medium, and 100 μL of complexes were added per well. Cells were incubated for 18–24 h (or up to 48 h depending on readouts) prior to downstream analyses.

**Adenoviral transduction**

pCMECs were seeded to reach ~70–80% confluence at the time of transduction. Cells were transduced with adenoviral vectors encoding nuclear-targeted RNaseH1 (Ad–NLS-RNaseH1) or the corresponding control virus (Ad-Control) in complete endothelial growth medium. After incubation for 6 h, the medium was replaced with fresh complete medium, and cells were cultured for an additional 48-72 h before downstream assays.

**RNA isolation and RT-qPCR**

Total RNA was extracted from mouse tissues and cultured cells with TRIzol reagent (Invitrogen, 15596-018). cDNA synthesis was performed using the PrimeScript RT Reagent Kit with gDNA Eraser (Takara, RR047A).RT-qPCR was performed with TB Green Premix Ex Taq II (Takara, RR820A). Relative expression was normalized to 18S rRNA.
For absolute quantification, a standard curve was generated using a quantified plasmid DNA standard containing the target amplicon. Copy numbers were calculated based on the molecular weight of the standard, and serial 10-fold dilutions were prepared to cover the expected dynamic range. Ct values were interpolated from the standard curve to obtain absolute copy numbers, which were reported as copies per ng of input total RNA. All reactions were run with melting-curve analysis, and no-template and no-reverse-transcription controls were included to exclude contamination and genomic DNA carryover.

**Western blot**

Tissues and cells were lysed in ice-cold lysis buffer and clarified by centrifugation (12,000 × g, 20 min, 4°C). Protein (50 μg per lane) was separated by 12% SDS–PAGE and transferred to PVDF membranes (Bio-Rad). Membranes were blocked with 5% BSA for 1 h and incubated with primary antibodies overnight at 4°C, followed by HRP-conjugated secondary antibodies for 2 h at room temperature. Chemiluminescent signals were developed using ECL reagent (Millipore). β-actin/H3 served as the loading control, and band intensities were quantified by densitometry.

**ChIP assay**

Chromatin immunoprecipitation (ChIP) was performed using the SimpleChIP® Enzymatic Chromatin IP Kit (Cell Signaling Technology, 9003) according to the manufacturer’s instructions. pCMECs were cross-linked with 1% formaldehyde for 10 min at room temperature and quenched with glycine for 5 min. After washing with ice-cold PBS, cells were lysed and nuclei were isolated. Chromatin was digested with micrococcal nuclease and briefly sonicated to obtain DNA fragments of ~300–500 bp. Immunoprecipitation was carried out overnight at 4°C with anti-H3K4me3(CST, 9751), H3K27ac(CST, 8173), ISL1 (Santa Cruz, sc-390793 X), or with the kit-provided IgG as a negative control. 10% of chromatin was reserved as input. Protein A/G magnetic beads were added to capture immune complexes. Samples were treated with RNase A and proteinase K, and purified DNA was analyzed by qPCR.

**Dual luciferase reporter assay**

The *Tek* promoter region (WT and Mut) and the Tek-e1 promoter region were amplified by PCR and cloned into the pGL4.14 firefly luciferase reporter vector, and the full-length ISL1 coding sequence was amplified and cloned into the pCMV expression vector. HEK293T cells were co-transfected with pGL4.14-empty or pGL4.14-promoter constructs, pCMV-empty or pCMV-ISL1, and a Renilla luciferase control plasmid using Lipofectamine 3000 (Invitrogen, L3000015). Firefly and Renilla luciferase activities were measured using the Dual-Luciferase Reporter Assay System (Promega, E1910), and firefly luciferase activity was normalized to Renilla luciferase activity.

**Chromosome Conformation Capture**

3C assays were performed according to the instructions(Bes5006, BersinBio^TM^). Cells were cross-linked with 1% formaldehyde at room temperature for 10 min, and the reaction was quenched with 125 mM glycine for 5 min. After washing with PBS, cells were collected and resuspended in ice-cold cell lysis buffer (10 mM Tris-HCl, pH 8.0; 10 mM NaCl; 0.2% NP-40; protease inhibitors), incubated on ice for 10 min, and then subjected to Dounce homogenization and centrifugation to obtain nuclei. Nuclei were resuspended in 1× 3C buffer, SDS was added to a final concentration of 0.1%, and samples were incubated at 65 °C for 10 min to open chromatin, followed by addition of Triton X-100 to a final concentration of 1% and incubation at 37 °C for 15 min to neutralize SDS. Restriction enzyme was then added, and chromatin was completely digested at 37 °C overnight. On the following day, the reaction mixture was diluted to 7–10 mL per sample, keeping the DNA concentration below 3 ng/μL, and T4 DNA ligase (100 U/mL) was added; ligation was carried out at 16 °C for 4 h and further incubated for 30 min at room temperature to complete the ligation reaction. Proteinase K (200 μg/mL) was subsequently added, and cross-links were reversed by incubation at 65 °C overnight, followed by RNA removal with RNase A (100 μg/mL, 37 °C, 30 min) on the next day. DNA was purified by phenol–chloroform extraction and ethanol precipitation, and the purified DNA was analyzed by quantitative PCR.

As a control for ligation products, BAC clones were first digested overnight with 10 U of the same restriction enzyme, followed by ligation with 10 U T4 DNA ligase at 16 °C overnight. DNA was then purified by phenol–chloroform extraction and ethanol precipitation in the same manner and analyzed by quantitative PCR.

**DNA–RNA Immunoprecipitation (DRIP)**

DRIP–qPCR was performed according to the DRIP method described by Ginno et al. ^4^. A total of 1 × 10^7 cells were collected and resuspended in 500 μL of lysis buffer (10 mM Tris-HCl, pH 8.0; 1 mM EDTA; 0.5% SDS), followed by the addition of proteinase K (final concentration 1 mg/mL) and incubation at 55 °C overnight to fully digest proteins. Genomic DNA was then extracted by phenol–chloroform extraction and ethanol precipitation, and the DNA pellet was dissolved in 200 μL of 10 mM Tris-HCl (pH 7.5). The resulting DNA was subsequently digested with a mixture of restriction enzymes (EcoRI, XbaI, HindIII, SspI, and BsrGI; NEB) at 37 °C overnight to fragment it to a size suitable for DRIP analysis.

For DRIP, 10 μg of digested DNA were added to 500 μL of immunoprecipitation buffer (15 mM Tris-HCl, pH 7.5; 1 mM EDTA) together with 10 μL of S9.6 antibody, and the mixture was rotated at 4 °C overnight to enrich genomic DNA–RNA hybrids (R-loops). In parallel, an equal aliquot of digested DNA was pretreated with RNase H at 37 °C for 1 h before incubation with S9.6 antibody and used as a negative control for R-loop–dependent signals. On the next day, protein A/G magnetic beads were added and rotated at 4 °C for 2 h to capture the immune complexes, followed by 4–5 washes with immunoprecipitation buffer. The bead-bound DNA–RNA hybrids were then incubated in elution buffer containing proteinase K (1 mg/mL) at 55 °C for 45–60 min to elute the complexes and digest residual proteins, after which DNA was purified by phenol–chloroform extraction and ethanol precipitation. The purified DRIP DNA and the corresponding input DNA (1–5% of the starting DNA) were analyzed by quantitative real-time PCR.

**3‘RACE**

MI border-zone tissues were processed under RNase-free conditions. Total RNA or nuclear RNA was isolated using the PARIS kit according to the manufacturer’s instructions, and residual genomic DNA was removed by DNase I treatment. For transcripts lacking poly(A) tails or with low polyadenylation, RNA was first polyadenylated using E. coli poly(A) polymerase at 37°C for 10–30 min in the presence of excess ATP. The reaction was terminated with EDTA and the RNA was purified. 3′RACE-ready cDNA was then synthesized using an oligo(dT)-adapter primer. First-round PCR was performed using a gene-specific primer (GSP) together with a universal adapter primer (UPM/adapter primer) under the following cycling conditions: 98°C for 30 s; 25–35 cycles of 98°C for 10 s, 60–70°C for 15 s, and 72°C for 20–60 s; followed by a final extension at 72°C for 2 min. When necessary, a second round of nested PCR was performed using a nested GSP and a nested universal primer to increase specificity. PCR products were resolved on a 1.2% agarose gel, and bands of the expected size were gel-excised, purified, and subjected to cloning and Sanger sequencing.

**CUT&Tag and sequencing data processing**

CUT&Tag was performed using the Hyperactive™ In-Situ ChIP Library Prep Kit for Illumina (Vazyme Biotech, TD903–TD904) according to the manufacturer’s protocol. Briefly, cells were immobilized on concanavalin A–coated magnetic beads and permeabilized with digitonin. Bead-bound cells were incubated sequentially with primary antibodies against H3K4me3 (CST, 9751) or RPB1 (CST, 14958), an appropriate secondary antibody, and Hyperactive pA–Tn5 transposase. Antibody-tethered pA–Tn5 cleaved DNA in the vicinity of targeted chromatin and simultaneously inserted P5/P7 adapters. Libraries were amplified by PCR with P5/P7 primers, purified, and assessed on an Agilent 2100 Bioanalyzer (Agilent Technologies).

Libraries were sequenced on an Illumina NovaSeq 6000 platform to generate 150-bp paired-end reads. Raw reads were trimmed for adapters and low-quality bases using fastp and aligned to the Mus musculus reference genome (GRCm39) with Bowtie2. Peak calling was performed using SEACR with the “stringent” setting. Peak tracks were inspected in IGV. Peaks were annotated to genomic features and nearby genes using ChIPseeker. Motif enrichment was assessed using MEME-suite tools (MEME and DREME) with subsequent comparison to motif databases. Differential binding analysis between case and control groups was performed with DiffBind by constructing a consensus peak set, computing read counts per peak, and identifying differential sites using thresholds of p value < 0.05 and |log2(fold change)| > 1.

**Public human cardiac snRNA-seq data reanalysis**

Public human cardiac single-nucleus RNA-sequencing data from Kuppe et al. were reanalyzed using the processed Kuppe_All_snRNA.h5ad object. Author-provided quality-controlled nuclei, original UMAP coordinates, and cell_type_original annotations were retained without recomputing UMAP, reclustering, or redefining cell identities. Control samples and acute myocardial infarction samples collected within 7 days after infarction were included. Remote-zone samples were assigned as RZ, infarct-zone samples as IZ, and samples containing border-zone tissue were assigned as BZ. The final cohort included 14 samples, comprising 4 control, 3 RZ, 3 BZ, and 4 IZ samples. Cell identities were displayed according to the original cell_type_original annotation, and annotation consistency was assessed using a conservative panel of canonical cell-type marker genes. For KDM5 family expression analysis, raw counts from raw.x were extracted from nuclei in the selected samples, normalized to 10,000 counts per nucleus, and log-transformed using log1p. Expression of KDM5A, KDM5B, KDM5C, and KDM5D was summarized by anatomical region and original cell-type annotation. For each group, average log-normalized expression and the percentage of expressing nuclei were calculated. In dot plots, color indicates gene-wise scaled average expression, and point size indicates the percentage of expressing nuclei.

**Participants and ethics**

We conducted a nested case-control study at Fujian Provincial Hospital from February 2019 to September 2023. The study protocol was approved by the hospital ethics committee (K2017-12-025) and was conducted in accordance with the Declaration of Helsinki. All participants provided written informed consent. We included patients with stable angina (chronic coronary syndrome) who had at least one angiographically confirmed 100% occlusion. Stable angina was diagnosed according to the European Society of Cardiology guidelines^5^. Exclusion criteria included pregnancy, lactation, malignancy with less than one year life expectancy, chronic heart failure, cor pulmonale, immune system diseases, history of coronary artery bypass grafting, percutaneous coronary intervention within the last three months, intermittent claudication, and insufficient serum volume. A total of 402 eligible patients were enrolled. Blood samples were collected upon admission, stored in anticoagulant tubes, and centrifuged at 3000 × g for 15 minutes before being stored at -80°C for long-term preservation.

**Coronary angiography**

Coronary angiography was performed following current guidelines. Two experienced cardiologists reviewed all angiograms using the European Society of Cardiology’s lesion classification scheme ^6^. Discrepancies were resolved by a third reviewer^7^. The Rentrop score assessed collateral circulation as follows: 0 = none, 1 = filling of side branches only, 2 = partial filling of the epicardial segment, and 3 = complete filling. Patients were classified into poor (grade 0 or 1) or good (grade 2 or 3) CCV groups. For patients with multiple total coronary occlusions, the vessel with the highest collateral grade was selected for analysis.

**ELISA detection**

Serum sTIE2 concentrations in CTO patients were quantified using the Human TIE2 ELISA Kit (Proteintech, KE00242) according to the manufacturer’s instructions. Peripheral blood was collected, allowed to clot, and centrifuged to isolate serum, which was aliquoted and stored at −80°C until analysis; repeated freeze–thaw cycles were avoided. Serum samples were measured in duplicate and diluted as recommended by the kit.

**Supplementary Figures**

**Supplementary Figure 1**

**(A)** Flow-cytometric assessment of the purity of magnetically isolated cardiac endothelial cells. **(B)** Quantification of histone-modification immunoblots corresponding to Fig. 1G (n = 6 per group; one-way ANOVA using Tukey’s multiple comparisons test). **(C)** Feature plots showing the expression of H3K4 methylation-related enzymes, including KMT2A, KMT2B, KMT2F, KMT2G, KDM5A, KDM5B, KDM5C, and KDM5D, in the human cardiac snRNA-seq dataset. **(D)** Dot plot showing the expression of KMT2 family members in endothelial nuclei from the RZ and BZ. **(E)** Quantification of KDM5A protein levels corresponding to Fig. 1K (n = 6 per group; one-way ANOVA using Tukey’s multiple comparisons test). **(F)** RT-qPCR analysis of Kdm5a mRNA levels in magnetically separated leukocytes, cardiac endothelial cells (cECs), and other cardiac cell fractions the RZ and BZ at day 3 after MI (n = 6 per group; unpaired two-tailed Student’s *t*-test). **(G–K)** Immunoblot analysis of KDM5A and H3K4me3 in leukocytes **(G)**, cardiac endothelial cells (H), and other cardiac cell fractions (I) isolated from the RZ and BZ at day 3 after MI, with quantification of KDM5A **(J)** and H3K4me3 **(K)** protein levels (n = 6 per group; unpaired two-tailed Student’s *t*-test). The data were presented as the means ± SD of at least three independent biological experiments.

**Supplementary Figure 2
(A)** Quantification of KDM5A, TIE2, and H3K4me3 protein levels corresponding to the immunoblots shown in **Fig. 3G** in border-zone endothelial cells isolated from *Cdh5*^Cre^, *Kdm5a*^cKO^, and KDM5A-IN-1-treated *Cdh5*^Cre^ mice at 3 days after MI (n = 6 per group; one-way ANOVA with Tukey’s multiple-comparisons test). **(B)** Immunoblot analysis and quantification of TIE2, phosphorylated and total PI3K, AKT, and eNOS, and VE-cadherin in primary cardiac microvascular endothelial cells isolated from *Cdh5*^Cre^ and *Kdm5a*^cKO^ mice and treated with vehicle or recombinant ANGPT1. (n = 6 per group; two-way ANOVA with Tukey’s multiple-comparisons test). Data are presented as mean ± SD. Each dot represents an independent biological replicate.

 **Supplementary Figure 3**

**(A)** RT–qPCR analysis of *Hoxb4, Lhx3, Nfe2l1, Isl1*, and *Pdx1* expression in endothelial cells transfected with the corresponding targeting siRNAs or negative-control siRNA (n = 6 per group; unpaired two-tailed Student’s t-test). **(B)** Genome-browser tracks showing H3K4me3 CUT&Tag enrichment across the *Isl1* locus in *Cdh5*^Cre^ and *Kdm5a*^cKO^ endothelial cells. **(C)** H3K4me3 ChIP–qPCR analysis at the *Isl1* locus in *Cdh5*^Cre^ and *Kdm5a*^cKO^ endothelial cells (n = 4 per group; unpaired two-tailed Student’s t-test). **(D)** RT–qPCR analysis of *Isl1* mRNA expression in *Cdh5*^Cre^ and *Kdm5a*^cKO^ endothelial cells (n = 6 per group; unpaired two-tailed Student’s t-test). **(E)** Immunoblot analysis of ISL1 in cytoplasmic and nuclear fractions from *Cdh5*^Cre^ and *Kdm5a*^cKO^ endothelial cells, with quantification of the nuclear-to-cytoplasmic (N/C) ISL1 ratio (n = 4 per group; unpaired two-tailed Student’s t-test). Data are presented as mean ± SD. Each dot represents an independent biological replicate.

**Supplementary Figure 4**

**(A)** Sliding-window analysis of GC content and GC skew across the Tek-e2 sequence. **(B)** DRIP–qPCR analysis of R-loop enrichment at the *Tek* intronic control region and two distal genomic loci on chromosomes 19 and 10 in endothelial cells treated with vehicle or Tek-e1 (n = 4 per group; unpaired two-tailed Student’s t-test). **(C)** S9.6 dot-blot analysis of global DNA hybrid abundance in vehicle- and Tek-e1-treated endothelial cells with or without RNase H1 treatment, with quantification of S9.6 signals normalized to total DNA staining (n = 4 per group; two-way ANOVA with Tukey’s multiple-comparisons test). **(D)** Immunoblot analysis and quantification of γH2AX levels normalized to total H2AX in endothelial cells treated with vehicle or Tek-e1 (n = 6 per group; unpaired two-tailed Student’s t-test). **(E)** Comparative genomic analysis of the mouse *Tek* intronic enhancer and its homologous region in human *TEK* intron 1, showing two LASTZ alignment blocks with 78.1% sequence identity (749/959 aligned bases). **(F)** Genome-browser tracks of the human region homologous to the mouse *Tek* intronic enhancer, showing Ensembl enhancer and ENCODE cCRE annotations, bidirectional CAGE signals, and H3K27ac and H3K4me3 enrichment in primary human endothelial cells.Data are presented as mean ± SD. Each dot represents an independent biological replicate.

 **Supplementary Figure 5
(A)** TTC staining of serial heart sections from *Cdh5*^Cre^ and *Kdm5a*^cKO^ mice at 12 h after MI. **(B–D)** Quantification of heart rate **(B)**, left ventricular internal diameter at end-diastole **(C)**, and left ventricular internal diameter at end-systole **(D)** at baseline and on days 3, 7, 14, and 28 after MI (n = 10 mice per group; two-way ANOVA with Sidak’s multiple-comparisons test). **(E)** Flow-cytometric gating strategy and Ki67 histograms in viable CD45^−^CD31^+^ cardiac endothelial cells on day 3 after MI, with quantification of Ki67 mean fluorescence intensity (n = 6 mice per group; unpaired two-tailed Student’s t-test). TTC, triphenyltetrazolium chloride; LVIDd, left ventricular internal diameter at end-diastole; LVIDs, left ventricular internal diameter at end-systole; MFI, mean fluorescence intensity; FMO, fluorescence minus one. Data are presented as mean ± SD. Each dot represents an independent mouse.

**Supplementary Figure 6**

*Cdh5*^Cre^ mice were treated with vehicle, whereas *Kdm5a*^cKO^ mice received vehicle or Compound 5 (25 mg/kg, i.p.) every 2 days on days 0, 2, 4, and 6 after MI. **(A, B)** Immunoblot analysis **(A)** and quantification **(B)** of TIE2, phosphorylated and total PI3K, AKT, and eNOS, and VE-cadherin in cardiac endothelial cells isolated from *Cdh5*^Cre^ mice treated with vehicle and *Kdm5a*^cKO^ mice treated with vehicle or the TIE2 kinase inhibitor Compound 5 on day 3 after MI (n = 6 per group; one-way ANOVA with Tukey’s multiple-comparisons test). **(C–F)** Analysis of NG2^+^ pericyte-covered microvessels **(C, E)** and VE-cadherin^+^ microvessels **(D, F)** in the infarct border zone on day 7 after MI (n = 6 per group; one-way ANOVA with Tukey’s multiple-comparisons test). **(G, I)** Analysis of 70-kDa FITC–dextran leakage in the infarct border zone on day 7 after MI (n = 6 per group; one-way ANOVA with Tukey’s multiple-comparisons test). **(H, J)** Analysis of microvessels occluded by 4-μm FITC-labeled carboxylated microspheres in the infarct border zone on day 7 after MI (n = 6 per group; one-way ANOVA with Tukey’s multiple-comparisons test). Data are presented as mean ± SD. Each dot represents an independent mouse.

**Supplementary Figure 7**

*Cdh5*^Cre^ mice were treated with vehicle, whereas *Kdm5a*^cKO^ mice received vehicle or Compound 5 (25 mg/kg, i.p.) every 2 days on days 0, 2, 4, and 6 after MI. **(A)** Late gadolinium enhancement magnetic resonance imaging in two-chamber long-axis and short-axis views and quantification of normalized enhancement in the infarct border zone on day 7 after MI (n = 6 per group; one-way ANOVA with Tukey’s multiple-comparisons test). **(B, C)** M-mode echocardiography **(B)** and quantification **(C)** of heart rate, ejection fraction, fractional shortening, left ventricular internal diameter at end-diastole, and left ventricular internal diameter at end-systole at baseline and on days 7 and 28 after MI (n = 10 per group; two-way ANOVA with Tukey’s multiple-comparisons test). **(D)** Masson’s trichrome staining of serial short-axis heart sections and quantification of infarct size and collagen volume fraction on day 28 after MI (n = 10 per group; one-way ANOVA with Tukey’s multiple-comparisons test). LVIDd, left ventricular internal diameter at end-diastole; LVIDs, left ventricular internal diameter at end-systole; LGE-MRI, late gadolinium enhancement magnetic resonance imaging; 2CH/LAX, two-chamber long-axis view; SAX, short-axis view. Data are presented as mean ± SD. Each dot represents an independent mouse.

**Supplementary Figure 8**

WT mice were treated with vehicle, Compound 5 (25 mg/kg), COMP-Ang1 (30 μg per mouse), or Compound 5 plus COMP-Ang1 via intraperitoneal injection every 2 days (days 0, 2, 4, and 6) following MI. All assessments were performed on day 7 after MI. **(A)** Immunoblot analysis and quantification of phosphorylated and total PI3K, AKT, and eNOS, and VE-cadherin in cardiac endothelial cells treated with vehicle, Compound 5, COMP-Ang1, or Compound 5 plus COMP-Ang1 (n = 6 per group; one-way ANOVA with Tukey’s multiple-comparisons test). **(B, C)** Analysis of NG2^+^ pericyte-covered microvessels **(B)** and VE-cadherin^+^ microvessels **(C)** in the infarct border zone (n = 6 per group; one-way ANOVA with Tukey’s multiple-comparisons test). **(D)** Analysis of 70-kDa FITC–dextran leakage in the infarct border zone (n = 6 per group; one-way ANOVA with Tukey’s multiple-comparisons test). **(E)** Analysis of microvessels occluded by 4-μm FITC-labeled carboxylated microspheres in the infarct border zone (n = 6 per group; one-way ANOVA with Tukey’s multiple-comparisons test). Data are presented as mean ± SD. Each dot represents an independent biological replicate or mouse, as appropriate.

**Supplementary Figure 9**

**(A)** Flow diagram of patient screening and enrollment in the CTO cohort. Of 493 consecutively screened patients, 402 were included in the final analysis and stratified according to Rentrop grade. **(B, C)** Systemic biodistribution of LNP@Cy5–Tek-e1 following intravenous administration on day 3 after MI. **(B)** Ex vivo fluorescence imaging of the liver, lung, brain, and kidney at 6, 12, 24, and 48 h after injection. **(C)** Quantification of fluorescence intensity in the indicated organs (n = 3 mice per time point; one-way ANOVA with Tukey’s multiple-comparisons test). **(D, E)** Assessment of liver function and systemic inflammatory responses in sham-operated mice receiving PBS and MI mice treated with PBS or LNP@Tek-e1. PBS or LNP@Tek-e1 was administered intravenously on days 3, 5, and 7 after MI (three injections). Blood samples were collected 4 h after the final injection on day 7 or 7 days after treatment cessation (day 14). **(D)** Serum ALT and AST levels. **(E)** ELISA analysis of serum IFN-α, IL-6, and TNF-α levels (n = 6 mice per group; one-way ANOVA with Tukey’s multiple-comparisons test). **(F)** Flow-cytometric gating strategy for identification of viable Cy5^+^ cells and CD45^−^CD31^+^ endothelial cells. **(G)** Flow-cytometric analysis of Cy5 fluorescence in cells isolated from the lung, kidney, and brain following treatment with LNP@empty or LNP@Cy5–Tek-e1. ALT, alanine aminotransferase; AST, aspartate aminotransferase; MI, myocardial infarction. Data are presented as mean ± SD. Each dot represents an independent mouse.

**Supplementary Figure 10**

Mice received three intravenous injections of LNP@empty or LNP@Tek-e1 on days 3, 5, and 7 after MI**. (A, B)** Analysis of NG2^+^ pericyte-covered microvessels **(A)** and VE-cadherin^+^ microvessels **(B)** in the infarct border zone on day 7 after MI (n = 6 mice per group; unpaired two-tailed Student’s t-test). **(C)** Analysis of 70-kDa FITC–dextran leakage in the infarct border zone on day 7 after MI (n = 6 mice per group; unpaired two-tailed Student’s t-test). **(D)** Analysis of microvessels occluded by 4-μm FITC-labeled carboxylated microspheres in the infarct border zone on day 7 after MI (n = 6 mice per group; unpaired two-tailed Student’s t-test). **(E)** Quantification of heart rate, fractional shortening, left ventricular internal diameter at end-diastole, and left ventricular internal diameter at end-systole at baseline and on days 7 and 28 after MI (n = 10 mice per group; two-way ANOVA with Sidak’s multiple-comparisons test). LVIDd, left ventricular internal diameter at end-diastole; LVIDs, left ventricular internal diameter at end-systole. Data are presented as mean ± SD. Each dot represents an independent mouse.

**Supplementary Table**

**Table S1.** Baseline Variables between poor collateral vessel Group and good collateral vessel Group

|  | **Poor collateral vessel (Rentrop score 0-1)** | **Good collateral vessel (Rentrop score 2-3)** | **p.value** |
| --- | --- | --- | --- |
|  | **n=150** | **n=252** |  |
| **Demographics** |  |  |  |
| Age, median (IQR), years | 65 (55-72) | 65 (58-72) | 0.570 |
| Sex, Female, n (%) | 22 (14.67%) | 40 (15.87%) | 0.856 |
| Systolic blood pressure, median (IQR), mmHg | 127 (113-140) | 127 (115-142) | 0.328 |
| Diastolic blood pressure, median (IQR), mmHg | 75 (66-80) | 77 (69-84) | 0.033 |
| Heart rate, median (IQR), beats/min | 77 (68-84) | 75 (68-84) | 0.501 |
| Body mass index, median (IQR), kg/m^2^ | 24.6 (23.2-26.9) | 24.0 (22.2-26.2) | 0.005 |
| **Clinical history** | | | |
| Prior myocardial infarction, n (%) | 21 (14.00%) | 31 (12.30%) | 0.736 |
| Current smoker, n (%) | 80 (53.33%) | 96 (38.10%) | 0.004 |
| Hypertension, n (%) | 117 (78.00%) | 157 (62.30%) | 0.002 |
| Diabetes, n (%) | 73 (48.67%) | 86 (34.13%) | 0.005 |
| Atrial fibrillation, n (%) | 9 (6.00%) | 17 (6.75%) | 0.933 |
| **Laboratory measurements** | | | |
| WBC, median (IQR), 10^9^/L | 7.10 (5.98-8.60) | 6.80 (5.71-8.40) | 0.237 |
| RBC, median (IQR), 10^12^/L | 4.52 (4.16-4.91) | 4.54 (4.11-4.84) | 0.683 |
| Platelets, median (IQR),10^9^/L | 217.50 (183.00-259.75) | 225.00 (183.50-270.00) | 0.570 |
| Total Cholesterol, mean ± SD, mmol/L | 4.39 ±1.30 | 4.27 ±1.29 | 0.363 |
| HDL-C, median (IQR), mmol/L | 0.90 (0.75-1.08) | 0.93 (0.79-1.15) | 0.085 |
| LDL-C, median (IQR), mmol/L | 2.65 (1.95-3.56) | 2.61 (1.90-3.38) | 0.600 |
| Serum creatinine mean ± SD, μmol/L | 102.25 ± 95.16 | 88.13 ± 40.16 | 0.086 |
| D-dimer, median (IQR), mg/L FEU | 0.30 (0.18-0.64) | 0.34 (0.15-0.82) | 0.635 |
| sTIE2, median [IQR], pg/mL | 1962.50 (1693.50-2331.25) | 2413.50 (1835.75-2990.50) | <0.001 |
| **Medical therapy during hospitalization** | | | |
| Aspirin use, n (%) | 145 (96.67%) | 242 (96.03%) | 0.958 |
| Statin use, n (%) | 147 (98.00%) | 242 (96.03%) | 0.387 |
| ACEI/ARB use, n (%) | 116 (77.33%) | 196 (77.78%) | 1.000 |
| β-blocker use, n (%) | 118 (78.67%) | 195 (77.38%) | 0.860 |
| Diuretic use, n (%) | 57 (38.00%) | 97 (38.49%) | 1.000 |
| **Echocardiography measurements** | | | |
| LVEF, mean ± SD, % | 55.93 ± 8.95 | 55.53 ± 9.65 | 0.680 |
| LVDd, mean ± SD, cm | 4.89 ± 0.65 | 4.89 ± 0.69 | 0.976 |
| LVPW,mean ± SD, cm | 0.98 ± 0.17 | 0.98 ± 0.15 | 0.972 |
| LVEDVi, mean ± SD, ml/1.73m^2^ | 98.71 ± 32.65 | 99.16 ± 37.92 | 0.901 |
| **Severity of CAD, n (%)** | | | 0.527 |
| 1 | 11 (7.33%) | 13 (5.16%) |  |
| 2 | 21 (14.00%) | 30 (11.90%) |  |
| 3 | 118 (78.67%) | 209 (82.94%) |  |

WBC, white blood cell- RBC, red blood cell- LDL-C, low-density lipoprotein cholesterol- HDL-C, high-density lipoprotein cholesterol- ACEI/ARB, angiotensin-converting enzyme inhibitor/angiotensin receptor blocker- LVEF, left ventricular ejection fraction- LVDd, left ventricular end-diastolic dimensions- LVPW, left ventricular posterior wall- LVEDVi, left ventricular end-diastolic volume index- CAD, coronary artery disease.

**Table S2. List of primers in the study**

| **Name** | **Primer sequence (5'-->3')** | **Application** |
| --- | --- | --- |
| *Tek*-F | GAGTCAGCTTGCTCCTTTATGG | RT-qPCR |
| *Tek*-R | AGACACAAGAGGTAGGGAATTGA | RT-qPCR |
| *Isl1*-F | GTAGAGGTGCAAAGTTACCAGCC | RT-qPCR |
| *Isl1*-R | TTAGAGCCTGGTCCTCCTTCTG | RT-qPCR |
| 18S rRNA-F | GCAATTATTCCCCATGAACG | RT-qPCR |
| 18S rRNA-R | GGCCTCACTAAACCATCCAA | RT-qPCR |
| *Kdm5a*-F | CACAGACCCGCTGAGTTTTAT | RT-qPCR |
| *Kdm5a*-R | CTTCACAGGCAAATGGAGGTT | RT-qPCR |
| *Kdm5b*-F | CTGGGAAGAGTTCGCGGAC | RT-qPCR |
| *Kdm5b*-R | CGCGGGGTGAAATGAAGTTTAT | RT-qPCR |
| *Kdm5c*-F | TTCAGGTTTACTCCCCGAATCC | RT-qPCR |
| *Kdm5c*-R | CCGCCGTTCTACATTGGGAAT | RT-qPCR |
| *Kdm5d*-F | CCAGGATCTGACGACTTTCTACC | RT-qPCR |
| *Kdm5d*-R | TTCTCCGCAATGGGTCTGATT | RT-qPCR |
| *Hoxb4*-F | GCACGGTAAACCCCAATTACG | RT-qPCR |
| *Hoxb4*-R | ACTCCTTCTCCAACTCCAGGA | RT-qPCR |
| *Lhx3*-F | GCAAGTGTCTCAAGTGCAGTG | RT-qPCR |
| *Lhx3*-R | TCCCGAAGCGCTTAAAGAAGT | RT-qPCR |
| *Nfe2l1*-F | GACTCGGGGTTCTCTGGAAAC | RT-qPCR |
| *Nfe2l1*-R | TTCTTTGGCTGGAGCTACAGG | RT-qPCR |
| *Pdx1*-F | CTCCCTTTCCCGTGGATGAAA | RT-qPCR |
| *Pdx1*-R | AATTCCTTCTCCAGCTCCAGC | RT-qPCR |
| Tek-e1-GSP | AGTGGGAGGAGAGTAAGATGCAGGCACC | 3′-RACE |
| Tek-e2-GSP | CACTTCCACACCATGACAGCCATGTCCC | 3′-RACE |
| Tek-e1-nested GSP | CAGGCACCAAGGTGACAGGCTCC | 3′-RACE |
| Tek-e2-nested GSP | CATGTCCCCATGGGTATTCACTCCC | 3′-RACE |
| *Tek* enhancer–promoter-F | GGCTGCTTTGACTGCAAAAGA | 3C-qPCR |
| *Tek* enhancer–promoter-R | CTTAACCCTCCCTGTGCTCAG | 3C-qPCR |
| *Tek* enhancer–intronic control-F | GGCTGCTTTGACTGCAAAAGA | 3C-qPCR |
| *Tek* enhancer–intronic control-R | TTTCAAGCCAAACACCCACTT | 3C-qPCR |
| *Tek*(0 to +200)-F | GGTCCTCATCGCATACCATACA | ChIP-qPCR |
| *Tek*(0 to +200)-R | GGGGCAGAGGGATCTTTGTTAC | ChIP-qPCR |
| *Tek*(−200 to 0)-F | AGGCGATCTGGGTGTAAGAAAA | ChIP-qPCR |
| *Tek*(−200 to 0)-R | GGCAATCAATTTGAGGGAGCG | ChIP-qPCR |
| *Tek*(−400 to −200)-F | CCTGTGCCAAGTTCTCCAACG | ChIP-qPCR |
| *Tek*(−400 to −200)-R | GCCCTGAGCCCAAATATACGC | ChIP-qPCR |
| *Tek*(−600 to −400)-F | TTCCATCACACGGGCTTTGA | ChIP-qPCR |
| *Tek*(−600 to −400)-R | CTCTTGGATTACTCCGGAGATTTTT | ChIP-qPCR |
| *Tek*(−800 to −600)-F | ACTCATCCATCACTCTGCTCC | ChIP-qPCR |
| *Tek*(−800 to −600)-R | CTGAAAATGTGCACCTCCCA | ChIP-qPCR |
| *Tek*(−1000 to −800)-F | CAAGACTAGCAGTGCCTCCG | ChIP-qPCR |
| *Tek*(−1000 to −800)-R | GCATGCCACTAGACACACGA | ChIP-qPCR |
| *Tek*(+6900 to +7400)-F | TCCAGCAGTCTGACCATGTTC | ChIP-qPCR |
| *Tek*(+6900 to +7400)-R | CCATCAGCAACAAATGTGCCA | ChIP-qPCR |
| *Isl1*(−400 to +100)-F | ACTTCTCAGCTCCAACTCAGC | ChIP-qPCR |
| *Isl1*(−400 to +100)-R | GATAATCAGAACAGCTGCGCC | ChIP-qPCR |
| Tek-e1 enhancer-F | GGCTTAGAGACAGGTGGCAAT | DRIP-qPCR |
| Tek-e1 enhancer-R | TCATTTGCTCTATCCCTGCCC | DRIP-qPCR |
| *Tek* intronic control-F | AGTGGGTGTTTGGCTTGAAAA | DRIP-qPCR |
| *Tek* intronic control-R | TGCAAATCAACTTTCCAAGCCT | DRIP-qPCR |
| chr19 similar locus-F | GCCTCTGAGGGACTGAAGAAC | DRIP-qPCR |
| chr19 similar locus-R | GAGGTAAGCTGTTCCCTTGCT | DRIP-qPCR |
| chr10 similar locus-F | TTTATGCAAAGTGTGGCGGTG | DRIP-qPCR |
| chr10 similar locus-R | TTTATTGCTGCTCTGGAGGCA | DRIP-qPCR |

**Table S3. List of siRNA in the study**

| **siRNA** | **Sense (5′→3′)** | **Antisense (5′→3′)** |
| --- | --- | --- |
| si-*Hoxb4* | GUAGAGAAGGGGAAAUAAACC | UUUAUUUCCCCUUCUCUACCC |
| si-*Lhx3* | GCAAAGACGACUUCUUUAAGC | UUAAAGAAGUCGUCUUUGCAG |
| si-*Nfe2l1* | GCCUGUAGAAGAAUUCAAUUU | AUUGAAUUCUUCUACAGGCUU |
| si-*Pdx1* | GGAAGAUAAGAAACGUAGUAG | ACUACGUUUCUUAUCUUCCUC |
| si-*Isl1* | GCGGCAAUCAAAUUCACGAUU | UCGUGAAUUUGAUUGCCGCUU |

**References**

1	Protti, A., Sirker, A., Shah, A. M. & Botnar, R. Late gadolinium enhancement of acute myocardial infarction in mice at 7T: cine-FLASH versus inversion recovery. *J Magn Reson Imaging* **32**, 878-886 (2010). <https://doi.org/10.1002/jmri.22325>

2	Schneider, J. E., Wiesmann, F., Lygate, C. A. & Neubauer, S. How to perform an accurate assessment of cardiac function in mice using high-resolution magnetic resonance imaging. *J Cardiovasc Magn Reson* **8**, 693-701 (2006). <https://doi.org/10.1080/10976640600723664>

3	Price, A. N. *et al.* Rapid assessment of myocardial infarct size in rodents using multi-slice inversion recovery late gadolinium enhancement CMR at 9.4T. *J Cardiovasc Magn Reson* **13**, 44 (2011). <https://doi.org/10.1186/1532-429x-13-44>

4	Ginno, P. A., Lott, P. L., Christensen, H. C., Korf, I. & Chédin, F. R-loop formation is a distinctive characteristic of unmethylated human CpG island promoters. *Mol Cell* **45**, 814-825 (2012). <https://doi.org/10.1016/j.molcel.2012.01.017>

5	Valgimigli, M. *et al.* 2017 ESC focused update on dual antiplatelet therapy in coronary artery disease developed in collaboration with EACTS: The Task Force for dual antiplatelet therapy in coronary artery disease of the European Society of Cardiology (ESC) and of the European Association for Cardio-Thoracic Surgery (EACTS). *Eur Heart J* **39**, 213-260 (2018). <https://doi.org/10.1093/eurheartj/ehx419>

6	Corrigendum to: 2019 ESC Guidelines for the diagnosis and management of chronic coronary syndromes. *Eur Heart J* **41**, 4242 (2020). <https://doi.org/10.1093/eurheartj/ehz825>

7	Rentrop, K. P., Cohen, M., Blanke, H. & Phillips, R. A. Changes in collateral channel filling immediately after controlled coronary artery occlusion by an angioplasty balloon in human subjects. *J Am Coll Cardiol* **5**, 587-592 (1985). <https://doi.org/10.1016/s0735-1097(85)80380-6>
